# Fast Label‐Free Metabolic Profile Recognition Identifies Phenylketonuria and Subtypes

**DOI:** 10.1002/advs.202305701

**Published:** 2024-02-13

**Authors:** Haiyang Su, Huiwen Zhang, Jiao Wu, Lin Huang, Mengji Zhang, Wei Xu, Jing Cao, Wanshan Liu, Ning Liu, Hongwei Jiang, Xuefan Gu, Kun Qian

**Affiliations:** ^1^ Henan Key Laboratory of Rare Diseases Endocrinology and Metabolism Center The First Affiliated Hospital, and College of Clinical Medicine of Henan University of Science and Technology Luoyang 471003 P. R. China; ^2^ State Key Laboratory of Systems Medicine for Cancer School of Biomedical Engineering Institute of Medical Robotics and Shanghai Academy of Experimental Medicine Shanghai Jiao Tong University Shanghai 200030 P. R. China; ^3^ Xinhua Hospital School of Medicine Shanghai Jiao Tong University Shanghai 200092 P. R. China; ^4^ Country Department of Clinical Laboratory Medicine Shanghai Chest Hospital Shanghai Jiao Tong University Shanghai 200030 P. R. China; ^5^ State Key Laboratory for Oncogenes and Related Genes Division of Cardiology Renji Hospital, School of Medicine, Shanghai Jiao Tong University Shanghai 200127 P. R. China; ^6^ School of Electronics Information and Electrical Engineering Shanghai Jiao Tong University Shanghai 200240 P. R. China

**Keywords:** diagnosis, mass spectrometry, metabolic profile, phenylketonuria, subtype identification

## Abstract

Phenylketonuria (PKU) is the most common inherited metabolic disease in humans. Clinical screening of newborn heel blood samples for PKU is costly and time‐consuming because it requires multiple procedures, like isotope labeling and derivatization, and PKU subtype identification requires an additional urine sample. Delayed diagnosis of PKU, or subtype identification can result in mental disability. Here, plasmonic silver nanoshells are used for laser desorption/ionization mass spectrometry (MS) detection of PKU with label‐free assay by recognizing metabolic profile in dried blood spot (DBS) samples. A total of 1100 subjects are recruited and each DBS sample can be processed in seconds. This platform achieves PKU screening with a sensitivity of 0.985 and specificity of 0.995, which is comparable to existing clinical liquid chromatography MS (LC‐MS) methods. This method can process 360 samples per hour, compared with the LC‐MS method which processes only 30 samples per hour. Moreover, this assay enables precise identification of PKU subtypes without the need for a urine sample. It is demonstrated that this platform enables high‐performance and fast, low‐cost PKU screening and subtype identification. This approach might be suitable for the detection of other clinically relevant biomarkers in blood or other clinical samples.

## Graphical abstract

The plasmonic silver nanoshells constructed microarrays are used for laser desorption/ionization mass spectrometry (MS) detection of phenylketonuria (PKU) by recognizing metabolic profile in dried blood spot (DBS) samples. The platform achieves precise PKU diagnosis and subtype identification in a single DBS test with a fast, label‐free, and low‐cost assay, comparable to existing clinical liquid chromatography MS methods.

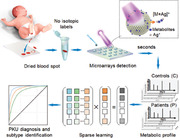

## 1 Introduction

The United Nations Population Division projects that the global prevalence of inherited metabolic diseases (IMDs) per year will increase from 100 000 cases in 1950 to over 140 000 by 2040, due to the growth of the global population and the number of newborns.^[^ ^1^ ^]^ Common IMDs amount to many species, with an accumulative incidence of ≈1/1000, resulting in irreversible mental retardation, physical handicaps, and even fatality to newborns.^[^ ^1^, ^2^ ^]^ Phenylketonuria (PKU) is a typical IMD,^[^ ^3^ ^]^ and screening for PKU now encompasses almost every newborn in developed countries.^[^ ^2^, ^4^ ^]^ Although PKU can be discovered in newborns, approximately half of the newborns are not screened in developing countries.^[^ ^4^, ^5^ ^]^ Concurrently, two subtypes of PKU (phenylalanine hydroxylase (PAH) and tetrahydrobiopterin (BH4) deficiency), call for different medical treatments including a phenylalanine‐restricted diet for PAH deficiency and medication therapy for BH4 deficiency.^[^ ^3^, ^6^ ^]^ Since the first‐generation screening by biochemical methods (e.g., Guthrie test and fluorometric/colorimetric assays), the second‐generation screening by liquid chromatography‐mass spectrometry (LC‐MS) has become the primary blood screening platform using dried blood spot (DBS) due to the accuracy and throughput.^[^ ^7^ ^]^ However, blood screening using DBS is limited to differentiate the subtypes of PKU, while highly time‐consuming and labor‐intensive urine tests (e.g., by high‐performance liquid chromatography, HPLC) are required to recall the newborns and select medical treatments.^[^ ^5^, ^6^ ^]^ A key difficulty with current PKU care is that screening platforms are slow and costly, delaying follow‐up diagnosis and making this essential platform inaccessible to global regions with limited resources.

Besides the fact that existing platforms cannot meet current diagnostic needs, tailored medical treatment for PKU critically relies on faster screening tools than those offered by existing platforms.^[^ ^6^, ^8^ ^]^ Highly promising clinical studies indicate that a phenylalanine‐restricted diet or medication therapy can control the pathological process of PKU.^[^ ^2^, ^6^, ^9^ ^]^ These ground‐breaking approaches have the potential to protect the nervous system, given they move toward large‐scale application in the patient population.^[^ ^6a^ ^]^ However, preliminary results of these studies show that restrictive diets and therapeutic interventions are most effective when implemented immediately following the newborn screening, with delays as short as hours to days potentially affecting efficacy.^[^ ^6^, ^10^ ^]^ The existing platforms for screening PKU and its subtypes are incapable of meeting these important emerging requirements, which could prevent newborns with PKU from receiving the optimum benefit from their medical treatments. Despite the obvious clinical need, developing next‐generation screening tools has been surprisingly challenging. In the past decades, numerous groups have demonstrated that the single common platforms, including biochemical methods and LC‐MS, fail to afford the clinically essential sensitivity and specificity when applied alone to both PKU screening and subtype identification.

The detection of metabolites as blood/urine biomarkers (including phenylalanine (Phe), tyrosine, and pterins) in a newborn with hyperphenylalaninemia is diagnostic of PKU and distinguishes the disease subtypes.^[^ ^3^, ^6^, ^11^ ^]^ Screening for metabolic disorders affecting the nervous system in PKU blood was first described in 1963 using a bacterial inhibition assay on DBS of the newborn heel.^[^ ^7a^ ^]^ The methodology has progressed with the identification of the three primary metabolites related to metabolic disorders against the nervous system. Numerous international programs have been conducted with a focus on enhancing PKU metabolite measurements.^[^ ^7^, ^8^, ^12^ ^]^

Metabolites with neurotoxicity and concomitance are generally the first to arise in individuals with PKU and are typically the most challenging to detect. The detection of disease‐related metabolites has been beyond the reach of standard approaches due to the molecular abundance (down to pmol) and sample complexity (multiple components including salt, protein, metabolites, and cells) in biofluids when metabolites are ionized in MS platforms. Although improved relative sensitivity has been achieved using LC‐MS with a chromatographic column for enrichment and purification.^[^ ^7^, ^13^ ^]^ Still, the Health Technology Assessment Program demonstrated that LC‐MS had poor overall performance and high‐cost labeling for the detection of disease‐related metabolites.^[^ ^14^ ^]^ This method is still the de facto standard for detecting metabolite biomarkers of PKU, and currently, there is no validated label‐free assay available. Importantly, this method is time‐consuming and labor‐intensive and is incapable of providing results before a clinician recalls to initiate diet or therapy, thus limiting the patient's access to medical interventions.

Using recent advances in tailored nanomaterials for laser desorption/ionization mass spectrometry (LDI MS) detection,^[^ ^15^ ^]^ we developed a plasmonic silver nanoshell platform that overcomes the major obstacles to fast, label‐free, and precision screening of IMDs. This approach allows direct detection of PKU metabolites in ultralow volumes of DBS extract (≈1 µL), without any enrichment and purification, yielding results after a short time (≈10 s). We found that our platform has a sensitivity (0.985 for PKU screening and 1 for subtype identification) and specificity (0.995 for PKU screening and 1 for subtype identification) comparable to those of LC‐MS/HPLC. Moreover, this new assay provides metabolic profile recognition, permits multiplexing of disease‐related metabolites, detects subtypes of PKU via multiple Elastic Net machine learning of a single DBS test, is cost‐efficient, and, notably, affords a real‐time “sample‐in, answer‐out” platform.

## 2 Results

### 2.1 Silver Plasmonic Nanoshells Enable Direct Detection of Metabolites

To address the clinical need for a fast, sensitive, and low‐cost blood screening test for PKU, we first investigated LC‐MS and HPLC, commonly applied in clinical situations. Agreed to what has been reported by other groups, we were unable to achieve the required diagnostic performance in a fast and label‐free manner, using these platforms separately to detect blood/urine biomarkers (**Table** **1**).

**Table 1:** Comparison of LC‐MS, HPLC, and LDI MS.

Method^a)^	Sample	PKU screening	Subtype identification	With isotopic labels or not	Cost (per sample)	Time^b)^
LC‐MS	DBS	Yes	No	With isotopic labels	$50–100	2 min
HPLC	UFPS	No	Yes	With isotopic labels	≈$1	5 min
LDI‐MS	DBS	Yes	Yes	Label‐free	≈$3	10 s

^a)^ For LDI MS analysis, one DBS sample was detected five times and the MS afforded fast analytical speed with 2 s per detection (with 2000 laser shots at a pulse frequency of 1000 Hz; See details in Experimental Section). For LC‐MS and HPLC analysis, the methods are described in detail in the Experimental Section of Supporting Information.

^b)^ The time was calculated from when we got the extracts of DBS/urinary filter paper spots (UFPS) to when we obtained the MS spectra of metabolites. Considering the urine transport, subtype identification is often not completed within 1 week for current clinical methods. Notably, requiring only the DBS, our platform enables PKU screening and subtype identification, greatly reducing time consumption. Min and sec are short for minutes and seconds, respectively.

**Figure 1:**
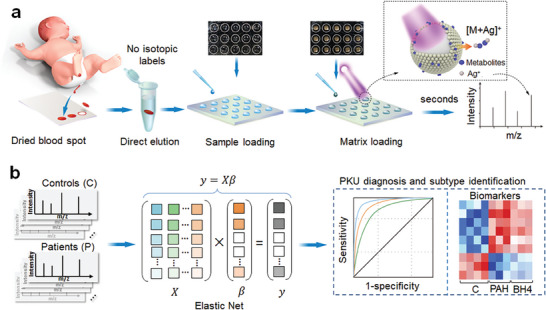
Overall schematics for extraction of DBS metabolic fingerprinting toward PKU diagnosis by sparse learning. a) Schematic workflow for the extraction of DBS metabolic patterns by SiO_2_@Ag assisted LDI MS, showing that 1 µL of extraction was consumed for direct analysis with Nd:YAG laser (355 nm) to record cation (Ag^+^) adducted signals of metabolites. b) Schematic diagram for sparse learning (by Elastic Net) of LDI MS results (*y*  =  *X*β) for PKU screening and subtype identification. Data matrix of DBS metabolic fingerprints was built through the processing of LDI MS results of controls (C) and patients (including phenylalanine hydroxylase (PAH) and tetrahydrobiopterin (BH4) patients) by sparse learning method, which was applied in five fold nested cross‐validations (20 rounds) for verification of receiver operating characteristics (ROC) curves and selection of biomarkers. The results included the differential metabolites of controls and PKU, as well as PAH and BH4.

**Figure 2:**
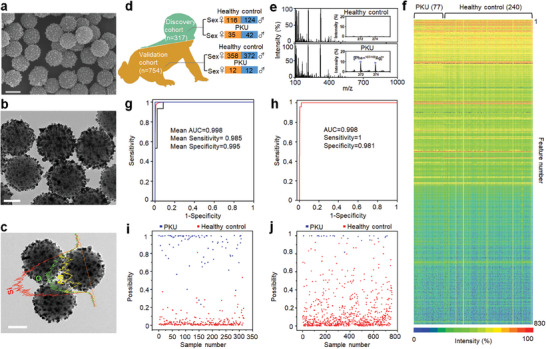
Characterization of SiO_2_@Ag nanoshells and metabolic profile recognition for PKU screening. Scanning electron microscope (SEM) image (a), transmission electron microscopy (TEM) image (b), and elemental distribution results (c) with oxygen (O) element in green, silicon (Si) element in red and silver (Ag) in yellow of SiO_2_@Ag nanoshells with nano‐crevices. Scale bar: 200 nm for SEM, 100 nm for TEM, and elemental mapping results. d) The gender distribution and number of discovery cohort (317) and validation cohort (754). e) Typical mass spectra of PKU patients and healthy controls. Inset: the signals at *m*/*z* of 271.98 and 273.98 were caused by silver ion‐adducted molecular peaks of phenylalanine (Phe). f) Heat map of DBS metabolic fingerprint for healthy controls (240) and PKU patients (77), based on 830 *m*/*z* features from each individual. g) Receiver operating characteristic (ROC) curve of Elastic Net as a classifier to discriminate PKU patients from healthy controls. The ROC curve was calculated by 20 rounds of fivefold nested cross‐validations with a mean AUC value of 0.998 with a 95% confidence interval (CI) of 0.985‐1 in the detection of PKU from healthy controls. ROC curves of the highest/averaged/lowest area under the curve (AUC) were shown in blue/red/black color, respectively. h) Validation result of 754 samples (healthy controls/PKU patients, 730/24) with an AUC value of 0.998 based on the optimized model. The DBS samples for the blind test were conducted from 754 individuals independent of the discovery cohort. All the spectra were recorded in the positive ion mode. Scatter diagram for PKU (blue square) and healthy controls (red square) from discovery cohort (i) and validation cohort (j).

We next applied nanotechnology to address the primary challenges of limited analytic speed and high cost (due to molecular abundance and sample complexity) that we encountered using other approaches. We constructed sample microarrays using nanostructured silver particles as the matrix in the LDI process (**Figure** **1**). The silver particles guarantee the generation of abundant nano‐crevices (≈4 nm range) agreeing with electron microscopy and elemental mapping results (**Figures** **2**a–c and S1, Supporting Information) that support size‐selective metabolite trapping and surface plasmon resonance to enhance LDI‐MS detection by ≈3 orders of magnitude (Figure S2a,b, Supporting Information). In addition, the plasmonic SiO_2_@Ag particles showed an absorption band (Figure S2c, Supporting Information) close to 355 nm (the wavelength for Nd:YAG laser used for LDI subsequently) and were negatively charged (Figure S2d, Supporting Information) in surface to facilitate the formation of outer cation layer and production of cation adduction signals.

To build a micro‐assay on this platform for DBS metabolite profiling, we directly loaded 1 µL of the sample (e.g., DBS extracts) and plasmonic silver nanoshells, probed it with Nd:YAG laser (to provide energy in LDI process), and then detected it with ionization through in situ cation adduction. The abundance of specific metabolite in the sample was reflected by the measured intensity at the given *m*/*z* (Figure S2e, Supporting Information). The LDI‐MS‐based metabolite detection for Phe, phenylacetaldehyde (Phen), or aminomalonic acid (AA) was first obtained on plasmonic silver nanoshells using a standard analyte solution containing known concentrations of the metabolite. We observed a sensitivity increase of 1000 orders of magnitude using the silver matrix compared with blank control (Figure S3, Supporting Information). Notably, we performed LDI MS detection, showing high salt tolerance in 0.5 m NaCl and protein endurance in 5 mg mL^−1^ bovine serum albumin (BSA), with a weight percentage of 1/100 000, demonstrating the high selectivity and consistency of the plasmonic silver matrix (Figure S4, Supporting Information). In contrast to the plasmonic SiO_2_@Ag nanoshells, smooth Ag or silica nanoparticles without nanoscale roughness and crevices on the surface for enhancing effect, resulted in lower sensitivity (Figure S5, Supporting Information). Other LDI‐MS matrices, such as organic matrices (alpha‐cyano‐4‐hydroxycinnamic acid (CHCA)/2,5‐dihydroxybenzoic acid (DHB)) or inorganic matrices (gold, magnetite particles), had much lower sensitivity and selectivity than the plasmonic silver matrix (Figure S6, Supporting Information). Particularly, the SiO_2_@Ag matrix exhibited more homogeneous matrix‐analyte crystallization than the commonly used organic matrix (with a coefficient of variations (CVs) of greater than 40%), leading to smaller CVs (less than 15%) for metabolite detection (Figure S7, Supporting Information). We also obtained the Ag‐adducted peaks of typical urea metabolite in urine and typical glucose metabolite in serum and plasma when SiO_2_@Ag nanoparticle was used as the matrix (Figure S8a, Supporting Information). However, we did not observe the adducted peaks of these typical metabolites when using organic matrices (Figure S8b,c, Supporting Information), demonstrating the universality of SiO_2_@Ag nanoparticles assisted LDI MS for sensitive metabolite detection in various clinical samples. Notably, the plasmonic silver platform was free of procedures like derivatization and chromatography, compared to LC‐MS for detecting disease‐related metabolites (Figure 1a and Table 1).

### 2.2 Label‐Free DBS Metabolic Profile Recognition for PKU Screening

In addition to direct detection with improved sensitivity, our approach offers several other advantages over LC‐MS (Table 1), including the ability to recognize the DBS metabolic profiles in a label‐free manner. LC‐MS analysis requires isotopes for labeling, making it inconvenient and expensive for newborn screening laboratories. However, the most speedy and efficient way to conduct screening is at the point of care such as a pediatrician's office. When the detection is fast and label‐free, a DBS sample may be detected simply and cheaply rather than requiring a newborn screening laboratory. This capability can fundamentally alter the current diagnostic approaches.

We constructed a platform combining the nano‐assisted LDI‐MS detection and sparse machine learning (Elastic Net), for the recognition of DBS metabolic profiles in a label‐free manner (Figure 1). To investigate the minimum sample number for machine learning statistically, we conducted a power analysis of ten samples (5/5, healthy control/PKU) as a preliminary study and got a predicted power of 0.90 with a sample number of 48 (24/24, healthy control/PKU) at a false discovery rate of 0.10 (Figure S9a, Supporting Information), proving that the statistics of machine learning results were at confidence level. There were two major components (inner loop and outer loop) in sparse machine learning, with a nested fivefold cross‐validation (CV) procedure and metabolic profiles (*X*) processed by sparsely constrained (β) for diagnosis (*y*), as shown in Figure 1b. For the inner CV loop, we included 254 individuals (80%) of the discovery cohort (317 individuals, including 240 healthy controls and 77 PKU patients) for five fold cross‐validation to determine the optimal hyper‐parameters. For the outer loop, we included 63 individuals (20%) of the discovery cohort (Figure 2d) to estimate the classification rate (Figure S9b, Supporting Information). The testing data of the outer loop were independent of the inner loop. To further validate our platform based on sparse machine learning, we built and tested the validation cohort (Figure 2d) with a new‐onset group (754 individuals including 730 healthy controls and 24 PKU patients). No significant difference in age and sex was observed between healthy control and PKU patients in the discovery and validation cohorts (*p* > 0.05, Table S1, Supporting Information).

Metabolites with a mass‐to‐charge (*m*/*z*) ratio of 100–1000 (Figure 2e) were measured using SiO_2_@Ag‐assisted LDI MS, consuming 1 µL of DBS eluent in several seconds. We can observe a clear difference between healthy control and PKU patients at *m*/*z* values 271.98 [Phe+^107^Ag]^+^ and 273.98 [Phe+^109^Ag]^+^ caused by the clinical biomarker Phe adducted Ag isotope peaks. Out of 100 000 signals extracted from the raw MS spectra, 830 features with the localized highest intensity for each sample were selected as a metabolic fingerprint for the disease classifier. For the discovery cohort, we built the heat map of the DBS metabolic fingerprint for healthy controls and PKU patients (PKU/healthy control, 77/240), showing signal uniformity for each individual (Figure 2f). After computer‐aided processing, we analyzed the area‐under‐the‐curve (AUC) with sensitivity and specificity to show diagnostic performance. We studied algorithms including Ridge, Naive Bayes, K‐Nearest Neighbor (KNN), and Elastic Net. As a result, for discovery cohort, Ridge exhibited average AUC of 0.887 with 95% confidence interval (CI) of 0.847–0.920 (sensitivity of 0.857 and specificity of 0.783), Naive Bayes afforded average AUC of 0.947 with 95% CI of 0.916–0.969 (sensitivity of 0.870 and specificity of 0.917) and KNN showed average AUC of 0.890 with 95% CI of 0.850–0.922 (sensitivity of 0.779 and specificity of 0.917), while Elastic Net afforded average AUC of 0.998 with 95% CI of 0.985‐1 (sensitivity of 0.985 and specificity of 0.995) (Figure 2g and Figure S10a and Table S2, Supporting Information), superior to other algorithms (*p* < 0.05 by DeLong test). The decision cut‐off of sensitivity and specificity (Figure S10a and Table S2, Supporting Information) produced by Ridge, KNN, and Naive Bayes was calculated by maximizing Youden index, while the sensitivity and specificity yielded by Elastic Net were calculated by looking for the point closest to (0, 1) corner in receiver operation curve (ROC) curve. We further re‐evaluated the sensitivity and specificity of different classification models by maximizing the Youden index, closest to the (0, 1) corner in the ROC curve, with equal sensitivity and specificity. As shown in Table S3, Supporting Information, Elastic Net yielded greater sensitivity and specificity than other models in each optimization criterion. Notably, we obtained the best specificity coupled with a high sensitivity by looking for the point closest to the (0, 1) corner in the ROC curve produced by Elastic Net.

Original metabolic features in the DBS presented high‐dimensional characteristics as shown in the experimental results (Figure 2f). However, not every feature had relevance to PKU disease, demonstrating the sparsity of the DBS metabolic profile comes from the sparsity of *m*/*z* features from MS data. Essentially, the KNN algorithm works by forming a majority vote between the K in most similar instances to a given test data.^[^ ^16^ ^]^ Naive Bayes classifier assumes that features are independent of each other, given their class variables, and also considers each of these features to contribute independently to the prediction.^[^ ^17^ ^]^ Therefore, KNN and Naive Bayes commonly do not yield sparse features. Ridge regression estimates the coefficients of models, shrinking the coefficients of correlated features equally toward zero. However, it does not force coefficients to zero, yielding limited sparse features, and hence cannot select a model with the most relevant subset of features.^[^ ^18^ ^]^ Notably, Elastic Net consists of L2 penalized Ridge regression and L1 penalized LASSO regression which forces many coefficients to be zero to obtain a sparse solution.^[^ ^19^ ^]^ This penalty term allows the model to select features as LASSO and also take care of correlated features as Ridge, providing a better model with reduced variance and improved interpretability.^[^ ^19^, ^20^ ^]^ Therefore, compared with Ridge, KNN, and Naive Bayes, Elastic Net was a sparse learning method that could remove redundant features and retain the most relevant features with respect to response samples, improving the classification accuracy.^[^ ^20^ ^]^ These results demonstrate that utilization of Elastic Net to analyze DBS metabolomics data is a potentially effective way for PKU screening.

With our platform, we further studied the performance of Elastic Net in the validation cohort (Figure 2h). Correspondingly, we got an AUC of 0.998 with a 95% CI of 0.992‐1 (sensitivity of 1 and specificity of 0.981) in the validation cohort, consistent with the outcome of the discovery cohort (Figure 2g). These results are comparable to the current PKU screening system (LC‐MS) (with a sensitivity of 0.95‐1 and specificity of 0.971‐1).^[^ ^21^ ^]^ Typically, in the scatter plot for the discovery cohort (Figure 2i) and validation cohort (Figure 2j), we can observe clear discrimination between PKU patients and healthy controls. The desirable performance of Elastic Net by our platform reveals that it will be practical to use computer‐aided diagnosis rather than routine manual diagnosis.

The above DBS samples of PKU patients were collected 10–60 days after birth. While the appropriate time for DBS sampling is 1 week after birth, the exact timing of the blood varies among countries/regions in real life due to the differences in medical conditions and territory of the countries.^[^ ^3a^ ^]^ Notably, the initial PKU screening by LC‐MS is usually characterized by a high false‐positive rate.^[^ ^21^ ^]^ Thus, clinicians have to recall the suspected patients for DBS re‐collection and testing, which is one reason why the DBS was collected at 10–60 days in this work. Besides, Xinhua Hospital is a well‐known newborn screening center, first carrying out PKU screening using tandem mass spectrometry in 2003 and promoting the popularity of the screening technology in China with a screening of ≈2 million newborns per year.^[^ ^22^ ^]^ The suspected PKU patients tested by other newborn screening centers usually come to Xinhua Hospital for further confirmation and subsequent therapy, which is another reason why newborn screening DBS was collected at 10–60 days of age. To further demonstrate our platform can also distinguish younger PKU patients from healthy controls, we re‐collected 15 DBS samples from younger PKU patients (DBS were collected within 2–5 days after birth, Table S4, Supporting Information). Although the elevation of Phe became more notable as the child got older, the *t*‐test showed a *p*‐value of 0.164 (Table S5 and Figure S11a, Supporting Information). The result demonstrated that there was no significant difference in the concentration of Phe between 10–60‐day‐old PKU and 2–5‐day‐old PKU. Furthermore, machine learning of metabolic profiles was conducted for the discrimination of PKU patients (2–5 days of age) and healthy controls, showing an AUC of 0.987 in the discovery cohort and an AUC of 1 in the validation cohort (Table S6 and Figure S11b, Supporting Information). The diagnostic performance was comparable to the results for the older PKU patients (10–60 days of age) detection. These results demonstrate that our platform enables precise PKU identification, unaffected by the age of patients.

### 2.3 Capability of Nano‐Platform is Equivalent to LC‐MS Plus HPLC

We collected DBS samples from individuals with PKU and healthy controls at Xinhua Hospital, Shanghai Jiao Tong University School of Medicine. We excluded subjects for whom informed consent was not granted or not possible. All newborns had routine LC‐MS‐based metabolic screening performed using DBS collected 2–60 days after birth, through the Shanghai Institute for Pediatric Research, Xinhua Hospital. We collected samples from 27 PKU patients (Table S7, Supporting Information) with subtype identification (13 were diagnosed with PAH deficiency and 14 with BH4 deficiency) and 20 healthy controls (Table S8, Supporting Information). In this work, all BH4 samples are 6‐pyruvoyl‐tetrahydropterin synthase (PTPS) deficiency. Globally, PTPS deficiency is the most frequent BH4 deficiency (≈54%), followed by dihydropteridine reductase (DHPR) deficiency (≈33%).^[^ ^23^ ^]^ However, in China, PTPS deficiency accounts for ≈96% of BH4 deficiencies, followed by DHPR deficiency (2.4%) and guanosine triphosphate cyclohydrolase I (GTPCH) deficiency (1.6%).^[^ ^24^ ^]^ Notably, the prevalence of BH4 deficiency is 3.8 per 1 000 000 live births in China.^[^ ^25^ ^]^ These reasons resulted in almost no BH4 samples with PTPS deficiency, GTPCH deficiency, and other types of BH4 deficiencies except for PTPS deficiency being collected in the hospital. In addition, DNAJC12 deficiency was recently found to be a new cause of PKU disease that was different from PAH and BH4 deficiencies.^[^ ^26^ ^]^ So far, we have not collected such samples. These are the reasons why we only included PAH deficiency and BH4 with PTPS deficiency as the experimental groups for PKU subtype identification.

**Figure 3:**
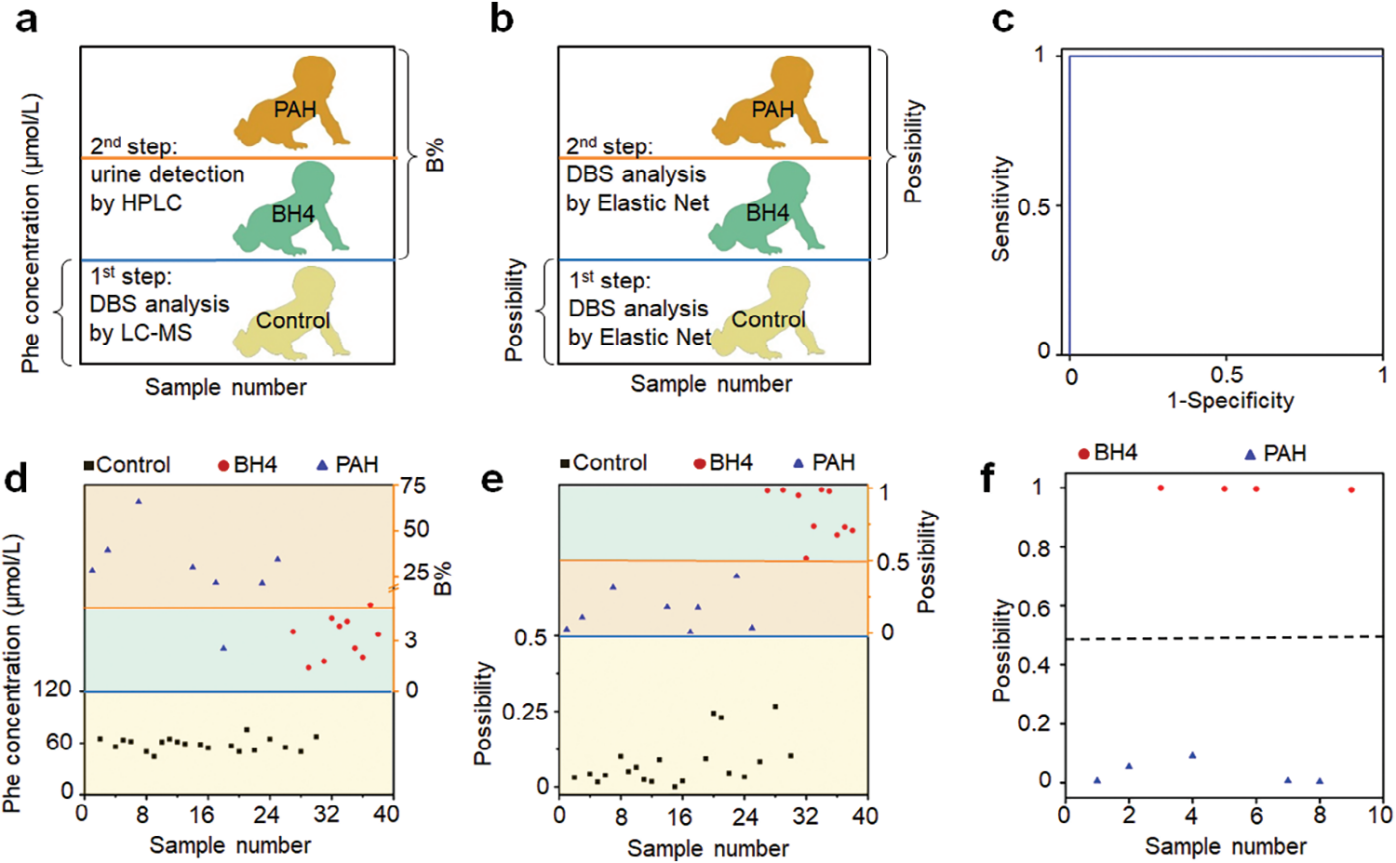
The discrimination of PAH and BH4 by sparse learning. a) Flow chart of PKU screening and subtypes identification by clinical methods. The first step (blue line) for PKU screening was realized by testing the Phe concentration (>120 µmol L^−1^). The second step (orange line) for subtype identification was finished by testing the percentage of biopterin ($B\%\;=\frac{\mathrm{biopterin}}{\mathrm{biopterin}+\mathrm{neopterin}}\;$) in the urine via HPLC. If B% was more than 5, the person was diagnosed with PAH, otherwise, judged as BH4. b) Flow chart of PKU screening and subtypes identification by sparse learning (Elastic Net) the DBS metabolic pattern. The first step (blue line) for PKU screening and the second step (orange line) for subtype identification were both realized by Elastic Net. c) ROC curve of Elastic Net as a classifier to discriminate PAH from BH4 in the discovery cohort. The result is from 20 rounds of five fold nested cross‐validations with a mean AUC value of 1 in the detection of 8 PAH and 10 BH4. Scatter diagram of control (black square, 20 samples), PAH (blue triangle, 8 samples), and BH4 (red circle, 10 samples) corresponding to d) clinical methods and e) Elastic Net. f) Scatter diagram of PAH (blue triangle, 5 samples) and BH4 (red circle, 4 samples) corresponding to the validation cohort. The possibility value of the dashed line is 0.5.

To establish a route for simultaneous PKU screening and subtype identification, we built a two‐step method performed by nano‐assisted LDI MS detection and Elastic Net for metabolic file recognition on a single platform (**Figure** **3**b). To facilitate a direct comparison of the described platform with the currently used standard that subjects had urine metabolic testing performed on a different HPLC platform to detect pterin for confirming PAH or BH4 deficiency after PKU screening via DBS analysis by LC‐MS (Figure 3a), we tested an aliquot of the same DBS samples used on the nano‐platform by LC‐MS in a clinical newborn screening laboratory for every PKU subject. The results of assays were compared to the ultimate clinical screening determined by gene screening according to genotype analysis and genotype validation combined with the expert judgment of the laboratory specialist or attending clinician.

As expected, all of the PKU patients tested positive by nano‐assisted LDI MS with Elastic Net (Figure 3e). The prediction probability for metabolic profile and scatter plot for subtypes identification by Elastic Net are plotted in Figure 3e,f. We found that nano‐assisted LDI MS afforded a sensitivity of 100% and specificity of 100% (Figure 3c,e) in the discovery cohort, for PKU screening equal to LC‐MS in the first step and for subtype identification superior to HPLC in the second step (Figure 3d,e). Excellent subtype diagnostic performance can also be achieved in the validation cohort (Figure 3f and Figure S10b, Supporting Information). We found that the LDI MS with Elastic Net was far superior on the plasmonic platform for the detection of metabolic profiles. Currently, pterins are still frequently analyzed in a urine sample for PKU subtype identification as the primary detection method such as in the USA and China.^[^ ^3^, ^25^ ^]^ Because the measurement of pterins in urine is more sensitive due to their higher concentrations in urine (with pterins concentration of ≈mmol mol^−1^ creatinine), in comparison to DBS (with pterins concentration of ≈nmol L^−1^) test.^[^ ^23^, ^27^ ^]^ However, the use of DBS instead of urine for analysis of pterins is easier to handle. Notably, our nano‐platform achieved both PKU diagnosis and subtype identification by one DBS test in this work, with the capability of being equivalent to LC‐MS plus HPLC.

### 2.4 Multiplexed Detection of Potentially New Biomarkers

**Figure 4:**
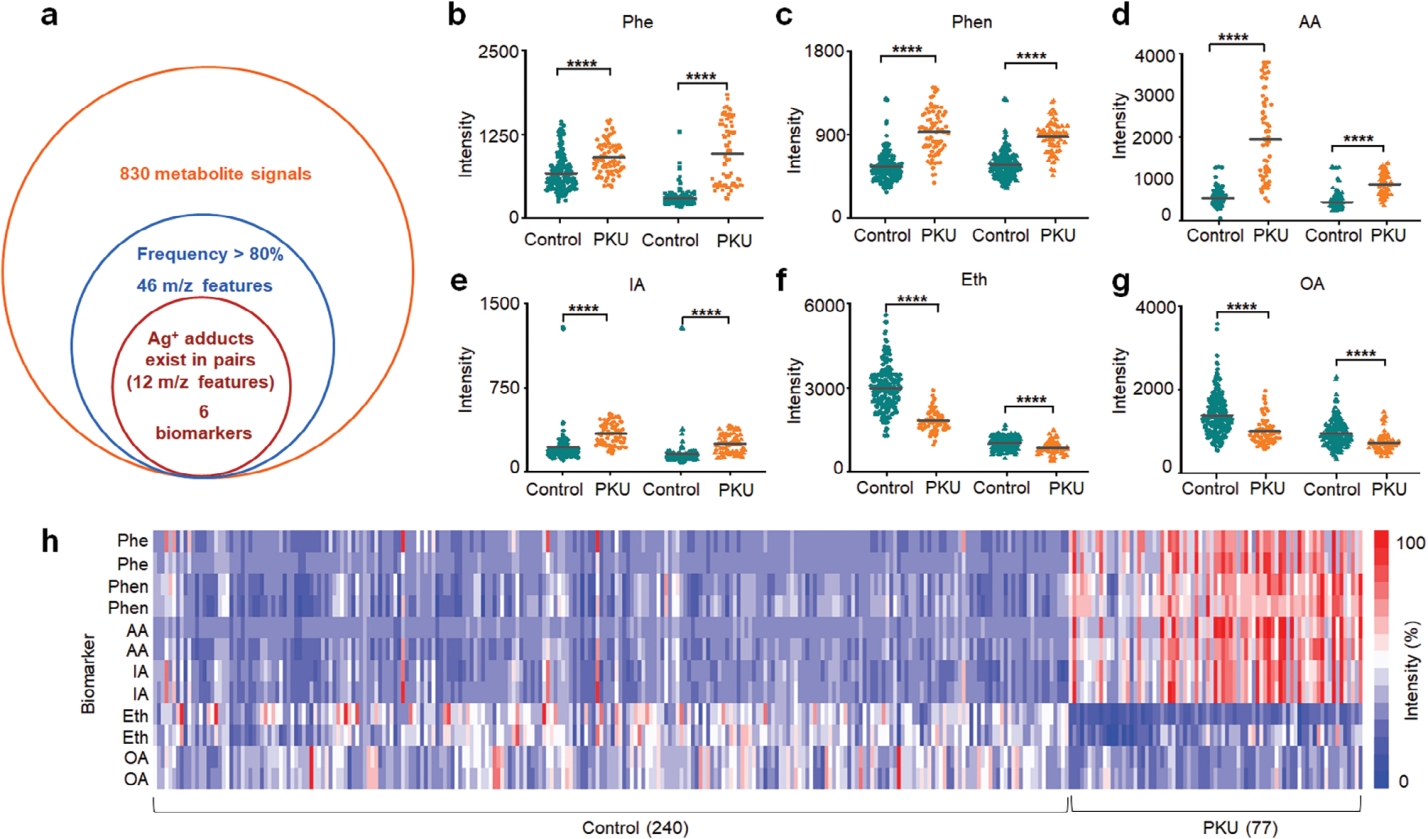
Differential biomarkers selected by sparse learning. a) Venn diagram of 46 *m*/*z* features with frequency of >80% selected by Elastic Net from 830 metabolite peaks in DBS for PKU screening (including 77 PKU patients and 240 healthy controls in the discovery cohort), 12 of which with Ag isotopes adducted signals in pairs, corresponding to 6 differential metabolic biomarkers. Scatter diagram of 6 key differential metabolites with [M+^107^Ag]^+^ and [M+^109^Ag]^+^ for PKU patients and healthy controls, including b) Phe, c) phenylacetaldehyde (Phen), d) aminomalonic acid (AA), e) indoleacrylic acid (IA), f) ethylamine (Eth), and g) oleic acid (OA). **** represented *p* < 0.0001.The circle represented the intensity of metabolites with [M+^107^Ag]^+^. The triangle represented the intensities of metabolites with [M+^109^Ag]^+^. h) Heat map of DBS metabolic fingerprinting for PKU patients and healthy controls, based on selected 6 biomarkers (12 *m*/*z* features).

Although modern LC‐MS favors Phe/Tyr detection in the targeted ion selection process/procedure, it cannot distinguish among PKU‐derived metabolites and needs HPLC for subtype identification. Blood metabolites are produced early in the absence of enzymatic function and are later excreted in urine as phenylpyruvic acid/pterins. Taking advantage of our platform to enhance the LDI MS of multiple metabolites with silver ion adduction, we obtained 830 metabolite signals in DBS for PKU screening, from which 46 *m*/*z* features with frequency of >80% (**Figure** **4**a). Further, we found 12 *m*/*z* features of Ag isotopes (with molecular weight 107 and 109, respectively) adducted signals in pairs with *p* < 0.05, corresponding six metabolic biomarkers (Figure 4b–g and Table S9, Supporting Information) and demonstrating the visible difference between PKU patients and healthy control (Figure 4h). We confirmed these metabolites according to standard references and the human metabolome database (http://www.hmdb.ca/) (Table S10, Supporting Information), including reported biomarkers (Phe, Phen, and oleic acid (OA)) for PKU screening and new biomarkers (AA, indoleacrylic acid (IA), and ethylamine (Eth)) annotated in the typical mass spectra (Figure S10c, Supporting Information). The metabolic profile of DBS was analyzed by untargeted detection, thus achieving the detection of multiple metabolites simultaneously. Sparse learning was used for the selection of disease‐related features and the construction of the classification model. Notably, the diagnostic performance (with AUC of 0.9998) of the classification model constructed by the 6‐biomarker panel outperforms every single biomarker (*p* < 0.05 by DeLong test, Figure S10d, Supporting Information). Therefore, the partial overlap of the single biomarker does not affect the diagnostic performance of PKU. Similarly, for subtypes, we identified three metabolic biomarkers (Figure S12 and Table S11, Supporting Information). We recorded metabolic profiles, including simultaneous detection of six subclasses of metabolites including amino acid (Phe), organic acid (AA, IA, OA and heptacosanoic acid), aldehydes (Phen), amine (Eth), lipid (cholest‐5‐ene), and vitamin (tetradecanoylcarnitine), for PKU screening and subtypes identification (Figure 4 and Figure S12, Supporting Information). This was done by probing the microarray spots of captured DBS metabolites from a patient's heel blood using plasmonic silver nanoshells assisted laser desorption and ionization.

Among the biomarkers selected by Elastic Net, Phe, Phen, AA, and IA showed elevated concentrations in PKU patients compared with healthy controls, while Eth and OA presented lower concentrations in patients. Especially, Phe and Phen were reported biomarkers whose accumulation was caused by the PAH or BH4 deficiency resulting in the thwarted conversion of phenylalanine to tyrosine.^[^ ^3^, ^28^ ^]^ Currently, Phe is the primary biomarker for PKU screening in clinical situations.^[^ ^3^, ^6^ ^]^ OA was also reported with an altered level in PKU patients.^[^ ^29^ ^]^ Although there is no direct evidence that AA, IA, and Eth are closely relevant to PKU disease, several studies demonstrated that altered AA levels were associated with neurologic disorders,^[^ ^30^ ^]^ while high Phe concentration damages the nervous system. IA is one of the indole acids that are usually present in increased amounts in PKU patients,^[^ ^31^ ^]^ and may be involved in the metabolism of the disease through conversion between indole acids. Eth is also an unreported biomarker related to PKU disease, however, the apparent absence of amines was found in patients with PKU.^[^ ^32^ ^]^

Of note, we selected the plasmonic silver particles to afford silver ion‐adducted signals for reporting the metabolic profile in DBS. This was the first time untargeted LDI MS metabolic microarrays were developed on a plasmonic silver substrate. Using this untargeted metabolic detection strategy, we identified PKU and subtypes that tested for Phe, Phen, AA, IA, Eth, and oleic acid against PKU and cholest‐5‐ene, tetradecanoylcarnitine, heptacosanoic acid against subtypes. Although further research is needed to interpret whether testing for these and other metabolites is clinically useful, this discovery exemplifies that the platform can be used as a new research tool that, unlike currently used panels, enables the direct testing of a set of potential biomarkers.

## 3 Discussion

Since the 1960s, it has been recognized that a fast and globally accessible low‐cost screening test for PKU would significantly advance the field by allowing on‐site diagnosis of newborns that require phenylalanine‐restricted diet and subtype identification for medication therapy.^[^ ^6^, ^7^ ^]^ In more recent years, this need has increased due to the increased number of newborns globally, and it appears that expeditious intervention with diet or drugs affects the efficacy of these treatments.^[^ ^3^, ^33^ ^]^ Despite this tremendous need, advancing the platform has proven to be remarkedly more difficult than that of many other disease screening technologies.

PKU is defined by the detection of one or a group of metabolites with neurotoxicity and concomitance.^[^ ^3^, ^6^, ^12^ ^]^ However, these biomarkers are difficult to identify and quantify from metabolic profiles in DBS. When present, these metabolites are found at concentrations in the micromolar to nanomolar range, and it is challenging to differentiate their signals from other metabolites and blood components. Many research groups have demonstrated that conventional metabolic assays using MS (isotopic) measurements, fail to detect metabolic biomarkers in a label‐free manner.^[^ ^7^, ^34^ ^]^ The relatively low abundance and high profile complexity when these assays are applied to measure disease‐related metabolites are the major causes of these failures.^[^ ^35^ ^]^ This is further confounded by the inherent challenge of ionizing the low molecular weight (MW) species with cation adduction from the whole profile of diverse molecules when using the key material as the matrix on a solid surface.^[^ ^36^ ^]^ The unique nano‐crevices of the plasmonic silver nanoshells permit the desorption and ionization of small metabolites while excluding large molecules. In addition, sparse machine learning (Elastic Net) maximizes biomarker selection while resisting nonspecific metabolites, resulting in label‐free recognition, and has a diagnostic sensitivity comparable to that of LC‐MS and HPLC.

The plasmonic silver nanoshells have excellent salt and protein endurance at low analyte concentrations (down to 10 ng µL^−1^, Figure S4, Supporting Information). LDI MS enhancement on plasmonic silver layers emanates from in situ size‐exclusive enrichment by the abundant nanoscale gaps in silver nanoshells and enhanced hot carrier production rates or LDI yield due to resonance emission dipole coupling to plasmonic modes. The LDI MS enhanced the cation‐adducted metabolite signals excluding nonspecific large molecules in situ.^[^ ^37^ ^]^ Combined with sparse machine learning, it is responsible for the direct label‐free protocol and lower limit of detection versus conventional matrices, leading to improved performance in PKU screening and subtype identification. To our knowledge, this is the first time that metabolite microarrays on plasmonic silver nanoshells have been used for newborn screening.

Furthermore, the ability to perform multiple machine learning enables our identification of different biomarker panels for PKU and subtypes. We are not aware of any previous report of untargeted metabolomic detection of these panels in a single assay platform for newborn screening, demonstrating how this platform can be used as a next‐generation research tool to test for putative novel metabolite biomarkers. Identifying biomarkers that can detect metabolic disorders in blood earlier than those currently in use would greatly advance the field by facilitating a series of biomedical and translation research opportunities.

Although LC‐MS and HPLC are the current standards for detecting PKU‐derived metabolites, these methods are cumbersome and not readily available in certain clinical settings.^[^ ^3^, ^34^ ^]^ First, LC‐MS requires complicated procedures, including isotopic labeling, derivatization, and chromatographic separation, when it is used for DBS detection to confirm the patient with elevated phenylalanine in the blood.^[^ ^12^, ^34^ ^]^ Noteworthy, these procedures are time‐consuming (≈1 h)^[^ ^7^, ^12^ ^]^ and costly ($50–100 per infant) (Table 1).^[^ ^38^ ^]^ Furthermore, the additional urine sample is commonly collected for PKU subtype identification through HPLC‐assisted biopterin analysis before developing treatment strategies.^[^ ^3^, ^25^ ^]^ The requirement of the additional urine sample and HPLC equipment further increases the cost and time consumption (20 days to 41 months).^[^ ^39^ ^]^ These two limitations often lead to the initiation of therapy cannot be achieved within 2 weeks, especially in resource‐poor areas and large countries where sample transport will take too much time. Even though the initial therapy can be completed within 2 weeks in some countries, the elevated phenylalanine in the blood would lead to neurological damage and mental retardation if the therapy is not given in the first days after birth.^[^ ^3^, ^40^ ^]^ Therefore, the development of rapid and inexpensive PKU screening methods is urgently needed. While biochemical assays are the standards for metabolite quantification and are widely used for clinical and translational purposes,^[^ ^3^, ^7^, ^41^ ^]^ they are inadequate for untargeted detection in PKU due to low analytical throughput caused by selective recognition of analytes by given enzymes or chemicals. In our work, the plasmonic silver platform allows label‐free identification of PKU and subtypes via a single LDI MS detection of DBS extracts without the requirement of additional urine collection and testing equipment. Our approach improves detection speed and reduces the cost to ≈$3 per infant (Table 1), taking into account the chemical consumption (e.g., particle synthesis, calibration standards, and solvents) and equipment depreciation (e.g., laser sources and detectors). Overall, our platform offers a fast, label‐free, and low‐cost method for the identification of PKU and subtypes, overcoming the costly and time‐consuming limitations caused by the current system of newborn screening.

Besides addressing the current clinical need for improved screening of PKU and subtypes, we believe that this technology will enable a broad range of advances in biomedical and translational research that were not previously feasible. For example, serial monitoring of metabolites in patients undergoing new interventions might predict efficacy that protects against continued nervous system destruction and may correlate with a change in metabolite concentration. Furthermore, following metabolite profiles at high resolution in patients considered high risk would yield great insight into the natural history of the development of metabolic diseases. Ultimately, we believe that this technology could be deployed to facilitate screening for diverse IMD‐derived metabolites, identifying those who would otherwise be at risk for progression to IMD, and testing preventative interventions before the onset of clinical symptoms.

## 4 Experimental Section

### Chemicals and Reagents

Ammonium hydroxide (28–30%), tetraethyl orthosilicate (TEOS, 96%), ethanol absolute (99.7%), silver nitrate (99.5%), tetrachloroauric acid tetrahydrate (HAuCl_4_·4H_2_O, 47.8%), hydrochloric acid (HCl, ≈36–38%), ethanol absolute (99.7%), sodium chloride (99.5%), FeCl_3_•6H_2_O (>97%), trisodium citrate (>99%), sodium acetate (>99%), methanol (99.8%), acetonitrile (99.5%), and ethylene glycol were purchased from Sinopharm Chemical Reagent Beijing Co., Ltd (Beijing, China). Polyvinylpyrrolidone (PVP, MW 40 kDa), phenylalanine (Phe, 98%), BSA, Pluronic F127, 2,5‐dihydroxybenzoic acid (DHB), alpha‐cyano‐4‐hydroxycinnamic acid (CHCA), IA (98%), and cholest‐5‐ene were ordered from Sigma‐Aldrich (St. Louis, MO, USA). Phenylacetaldehyde (Phen, 95%), OA (99%), and ascorbic acid (99%) were ordered from Aladdin Reagent Co., Ltd (Shanghai, China). AA (97%) was bought from Shanghai Macklin Biochemical Co., Ltd. Heptacosanoic acid (98%) was purchased from TCI Development Co., Ltd (Shanghai, China). [^2^H_5_]‐Phe and [^2^H_5_]‐Tyr was bought from Cambridge Isotope Labs (America). All aqueous solutions throughout the experiments were prepared with deionized water (18.2 MΩ·cm, Milli‐Q, Millipore, GmbH).

### Preparation of Silica Nanoparticles

Mono‐dispersed spherical silica particles were prepared as the core using the well‐known Stöber method.^[^ ^42^ ^]^ Briefly, 53 mL ethanol absolute, 2.33 mL H_2_O, and 2.1 mL ammonium hydroxide were mixed with 3 mL TEOS and stirred at 25 °C for 7 h. Then, the products were collected by centrifugation with three ethanol/water cycles at 10 000 rpm for 10 min. The resulting silica nanoparticles were dried in the oven at 60 °C before use.

### Preparation of SiO_2_@Ag Nanoparticles

SiO_2_@Ag core–shell particles were synthesized by coating silver nanoparticles on the surface of silica nanoparticles. Overall, the as‐prepared silica particles (0.3 g) were dispersed in ethanol absolute (15 mL). Then, freshly prepared [Ag(NH_3_)_2_]^+^ ion solution (0.59 m, 1.7 mL) was added to the above dispersion of particles and sonicated for 30 min. The dispersed particles were subsequently mixed with 50 mL of PVP ethanol solution (0.5 mm) and stirred at 70 °C for 7 h to conduct a silver mirror reaction. The silver mirror reaction was repeated three times to obtain SiO_2_@Ag core–shell nanoparticles.

### Preparation of Silver Nanoparticles

The freshly prepared [Ag(NH_3_)_2_]^+^ ion solution (0.59 m, 3.4 mL) was mixed with 40 mL of PVP ethanol solution (0.5 mm) and stirred at 70 °C for 7 h to conduct a silver mirror reaction. The final products were washed with water and ethanol three times, respectively. Then the products were vacuum‐dried at 60 °C.

### Preparation of Gold Nanoparticles

Gold nanoparticles were prepared as reported.^[^ ^37f^ ^]^ Briefly, 3.6 mL of H_2_O, 60 µL of HCl (6 m), and 60 mg of Pluronic F127 were mixed. After Pluronic F127 was completely dissolved, 3 mL of ascorbic acid solution (0.1 m) and 1.2 mL HAuCl_4_·4H_2_O solution (10 mm) were added to the above solution. The mixed solutions were sonicated in a water bath at 45 °C for 1 h. The obtained Au nanoparticles were collected by three washing/centrifugation cycles with ethanol and water, and following dried in an oven at 60 °C before use.

### Preparation of Magnetite Nanoparticles

The magnetite nanoparticles were prepared using a slightly modified solvo‐thermal method based on the article published.^[^ ^43^ ^]^ Briefly, 1.5 g of FeCl_3_•6H_2_O, 0.36 g of trisodium citrate, and 2.4 g of sodium acetate were mixed with 50 mL of ethylene glycol under vigorous stirring until completely dissolved. Then, the mixture was transferred into a Teflon‐lined stainless‐steel autoclave (with a capacity of 100 mL) for heating at 200 °C for 10 h. After that, the autoclave was taken out and cooled to room temperature. The as‐prepared products were washed with ethanol and deionized water five times, and finally vacuum dried at 60 °C.

### Material Characterization

Scanning electron microscope (SEM) images were recorded on an S‐4800 (Hitachi, Japan), by dropping ≈1 µL of water suspension of materials on the aluminum foil. Transmission electron microscope (TEM) images were carried out using a JEM‐2100F instrument (JEOL, Japan), by depositing the diluted colloidal suspension on a copper grid. Zeta potential measurements were performed on a Nano‐ZS90 instrument (Malvern, Worcestershire, UK) in water at room temperature.

### Study Design

The purpose of this study was to develop plasmonic silver nanoshells assisted LDI MS for simultaneous PKU screening and subtype identification on a single platform in a rapid and cost‐effective manner. The research subjects were recruited from Xinhua Hospital affiliated to Shanghai Jiao Tong University School of Medicine. All the investigation protocols were approved by the Institutional Ethics Committees of Xinhua Hospital and Shanghai Jiao Tong University, under the approved protocol #XHEC‐D‐2021‐152. All subjects provided informed consent to participate in the study and approved the use of their biological samples for analysis. All experiments were performed following institutional guidelines and in compliance with relevant laws. For DBS preparation, a sterile, disposable lancet was used to puncture the infant's heel in a down position at or below the heart level. Then, the first blood of drop was wiped away, and the second large blood drop was used to apply to the surface of the filter paper circle. The blood spots were dried horizontally for 3–4 h at ambient temperature and stored at 4 °C until further analysis. An automated testing and analysis platform for PKU screening and subtype identification can be built based on previous work in material synthesis and data processing.^[^ ^37^, ^44^ ^]^

### Study Population

First, 1085 subjects were recruited in Xinhua Hospital affiliated to Shanghai Jiao Tong University School of Medicine including 115 PKU patients (101 PAH and 14 BH4 patients) and 970 healthy controls. All newborns were granted informed consent and had routine LC‐MS‐based metabolic screening performed using DBS collected 2–60 days after birth. For PKU screening, 1071 subjects were randomly assigned to 1) a discovery cohort of 77 PKU patients and 240 healthy controls; 2) an independent validation cohort of 24 PKU patients and 730 healthy controls. Similarly, for PKU subtype identification, 27 subjects were randomly assigned to 1) a discovery cohort of 8 PAH and 10 BH4 patients; 2) an independent validation cohort of 5 PAH and 4 BH4 patients. The sex of PKU patients and healthy controls were matched with no significant difference for the discovery cohort, as well as of PAH patients and BH4 patients, by 𝜒2 test in the SPSS package (version 19.0, SPSS Inc., USA) respectively.

Second, the above DBS samples of PKU patients were collected 10–60 days after birth. To further demonstrate that the platform can also distinguish younger PKU patients from healthy controls, 15 DBS samples were re‐collected from younger PKU patients (DBS were collected within 2–5 days after birth).

### LDI MS for the Detection of Metabolites in Dried Blood Spot

For the detection of metabolites, standard small molecules (Phe, Phen, and AA) were dissolved by step‐wise dilutions with concentrations ranging from 100 to 1 ng µL^−1^. Standard molecules were mixed with salts (NaCl, 0.5 m) and proteins (BSA, 5 mg mL^−1^) to explore the detection efficiency of different matrices in high concentrations of salt and proteins.

For the detection of metabolites on the DBS, a 3.2 mm circle was first punched from the center and kept in a tube. Then, a mixture of solvents (H_2_O/methanol/acetonitrile, v/v/v = 2:1:1) was added to the tube and shook the tube on an orbital shaker. The sample was then centrifuged at 10 000 rpm for 10 min to remove the sediment. The retaining supernatant was collected for further analysis.

In a typical LDI MS experiment, SiO_2_@Ag nanoshells were dispersed in water at a concentration of 1 mg mL^−1^ for use as a matrix. 1 µL of analyte solution (either standard small molecules or DBS extract) was spotted on the polish plate and dried in air at room temperature. Then, 1 µL of matrix slurry was added and dried for LDI MS analysis. For LDI MS using other nanoparticles (e.g., SiO_2_, Ag, etc.) as matrices, the materials were dispersed in water at a concentration of 1 mg mL^−1^. Mass spectra were recorded on an AutoFlex TOF/TOF mass spectrometer (Bruker, Germany) equipped with an Nd:YAG laser (2 kHz, 355 nm). The acquisitions were performed in positive reflector ion mode employing delayed extraction with a repetition rate of 1000 Hz and an acceleration voltage of 20 kV. The delay time for this experiment was optimized to 250 ns. The number of laser shots was 2000 per analysis for all LDI MS experiments. Every DBS sample was detected five times and the MS afforded fast analytical speed with 2 s per detection.

### Classification Modeling

For a typical machine‐learning classification, five mass spectra obtained for each sample were used to build molecular databases. Pre‐processing of the raw mass spectra data, including baseline correction, peak detection, extraction, alignment, normalization, and standardization, was carried out by MATLAB (R2016a, The MathWorks, Natick, MA). After that, the pre‐processing MS data matrix with sample label (“0” for healthy control and “1” for PKU patients) was analyzed by different algorithms. Ridge, KNN, Naive Bayes, and Elastic Net algorithm was performed in MATLAB. All the classification models were set with five fold cross‐validation to estimate the performance of the predictor. The performance of the classifiers was evaluated based on the ROC by the AUC, calculating the proportions of concordant pairs among all pairs of observations, with 1 indicating perfect prediction accuracy.

For biomarker discovery of PKU screening, only the discovery cohort was used. For biomarker discovery of subtype identification, all 27 subjects, including 13 PAH and 14 BH4 patients, were used for data processing. Classification analysis was performed on the DBS metabolic profile by Elastic Net algorithm, using a “home‐built” code by MATLAB (R2016a, The Mathworks, USA). The custom computer codes utilized during the current study are available from the corresponding author upon reasonable request, due to the competing financial interests. For a valid comparison, the same experimental configuration with the preconstructed elastic net method was strictly followed. The classification performance was evaluated by a five fold CV (including inner loop and outer CV), with 20 rounds for each fold yielding 100 models in total. The ROC and AUC were generated for predicted responses on both the discovery and validation cohorts. To identify the metabolic signature that contributed the most to the classifier, *m*/*z* features were chosen according to 1) the model selected frequency with repeat occurrence over 80% in 100 models; 2) *p* < 0.05 according to two‐sided *t*‐test; and 3) co‐existence of ^107^Ag^+^‐ and ^109^Ag^+^‐adducted signals. The potential metabolic biomarkers were identified based on the cross‐comparison of reference standards and accurate mass from the online database (HMDB). The classification performance of Ridge, K‐Nearest Neighbor (KNN), and Naive Bayes was also evaluated by a five fold CV.

### LDI MS for the Detection of Metabolites in Serum, Plasma, and Urine

Before detection, serum and plasma were diluted with deionized water ten fold, and urine was diluted with deionized water five fold. After that, 1 µL of analyte solution (diluted serum, plasma, or urine) was spotted on the polish plate and dried in air at room temperature. Then 1 µL of matrix slurry (1 mg mL^−1^ of SiO_2_@Ag nanoshells, 10 mg mL^−1^ of CHCA or DHB (dissolved in 0.1% TFA solution (water/acetonitrile = 7/3, v/v)) was added and dried for LDI MS analysis. Mass spectra were recorded on an AutoFlex TOF/TOF mass spectrometer (Bruker, Germany) equipped with an Nd:YAG laser (2 kHz, 355 nm).

### LC‐MS for Dried Blood Spot Metabolites Analysis

For every DBS, a 3.2 mm circle was punched from the areas of DBS and placed into a 96‐well microtiter plate. Subsequently, deuterium‐labeled internal standard solutions (including 5 µmol L^−1^ each of [^2^H_5_]‐Phe and [^2^H_5_]‐Tyr) of 100 µL methanol were added and the 96‐well microtiter plate was gently shaken during the 30‐min extraction of the metabolites. Then, the extraction was transferred to a second 96‐well microtiter plate and dried at 55 °C in an N_2_ atmosphere. Butanol‐HCl (60 µL) was added into each sample well and the 96‐well microtiter plate was covered with a thin Teflon film, and placed in a 65 °C forced air oven for 15 min. After removing the plate from the oven, the butanol‐HCl was removed by blow‐drying at 55 °C. The derivatized samples were reconstituted in 100 µL of a mixture of 80% acetonitrile and 20% water. Each plate was covered with aluminum foil and then for LC‐MS (UPLC‐Xevo‐TQ/TQD, Waters, America) analysis.

### HPLC for Urinary Pterin Analysis

To confirm the subtype of PKU patients, urine filter paper spots were collected for urinary pterin analysis. The urine pterins were analyzed by HPLC using the Waters 2695 liquid chromatograph (WAT 270 886, Japan) following procedures.^[^ ^45^ ^]^ The analyzed pterins included biopterin (B), neopterin (N), and B%. B% was calculated by [B/(B+N)×100].

### Statistical Analysis

All the univariate analyses in this work were performed to calculate the *p*‐value for statistical demonstration, based on the SPSS software (version 19.0, SPSS Inc., Chicago).^[^ ^46^ ^]^ DeLong test was conducted on the Medcalc software (version 20.110, MedCalc Software Ltd, Belgium). *p* < 0.05 was considered statistically significant.

## Conflict of Interest

The authors declare competing financial interests. The authors have filed patents for both the technology and the use of the technology to detect bio‐samples.

## Author Contributions

H.S., H.Z., and J.W. contributed equally to this work. K.Q., X.G., H.J., and H.Z. conceptualized and designed the study. H.Z. also finished the acquisition of all DBS samples. H.S. performed most of the experiments and collected and analyzed MS data. L.H. and J.W. helped synthesize the SiO_2_@Ag nanoshells. J.W., M.Z., W.X., W.L., and N.L. helped process MS data. J.C. provided suggestions for the synthesis of nanoparticles and data processing. The manuscript was written by H.S. and revised by J.W., K.Q., X.G., H.J., and H.Z. All authors reviewed the final version of the manuscript. The authors also thank Dr. Chen Xi from Urumqi Maternal and Child Health Care Hospital for clinical data collection.

## Supporting information

Supporting Information

## Data Availability

The data that support the findings of this study are available from the corresponding author upon reasonable request.
