# Supplementary material for: Fast Label‐Free Metabolic Profile Recognition Identifies Phenylketonuria and Subtypes
Source: Adv Sci (Weinh). 2024 Feb 13;11(15):2305701. doi: 10.1002/advs.202305701 (PMC11022714; doi:10.1002/advs.202305701)
Supplement: Supplementary file 1 — Supporting Information [file ADVS-11-2305701-s001.pdf]

ADVANCED SCIENCE
Open Access

Supporting Information

for *Adv. Sci.*, DOI 10.1002/advs.202305701

Fast Label-Free Metabolic Profile Recognition Identifies Phenylketonuria and Subtypes

Haiyang Su, Huiwen Zhang, Jiao Wu, Lin Huang, Mengji Zhang, Wei Xu, Jing Cao, Wanshan Liu, Ning Liu, Hongwei Jiang, Xuefan Gu* and Kun Qian**

Supporting Information

Fast label-free metabolic profile recognition identifies phenylketonuria and subtypes

Haiyang Su†, Huiwen Zhang†, Jiao Wu†, Lin Huang, Mengji Zhang, Wei Xu, Jing Cao, Wanshan Liu, Ning Liu, Hongwei Jiang, Xuefan Gu*, Kun Qian**

H. S., H. J.
Henan Key Laboratory of Rare Diseases, Endocrinology and Metabolism Center, The First Affiliated Hospital, and College of Clinical Medicine of Henan University of Science and Technology, Luoyang, China, 471003.
Email: jianghw@haust.edu.cn (H. J.)

H. Z., X. G.
Xinhua Hospital, School of Medicine, Shanghai Jiao Tong University, Shanghai, 200092, P. R. China.
Email: gu_xuefan@163.com (X. G.)

H. S., J. W., M. Z., J. C., W. L., K. Q.
State Key Laboratory of Systems Medicine for Cancer, School of Biomedical Engineering, Institute of Medical Robotics and Shanghai Academy of Experimental Medicine, Shanghai Jiao Tong University, Shanghai 200030, P. R. China
Email: k.qian@sjtu.edu.cn (K. Q.)

L. H.
Country Department of Clinical Laboratory Medicine, Shanghai Chest Hospital, Shanghai Jiao Tong University, Shanghai, 200030, P. R. China.

W. X.
State Key Laboratory for Oncogenes and Related Genes, Division of Cardiology, Renji Hospital, School of Medicine, Shanghai Jiao Tong University, 160 Pujian Road, Shanghai, 200127, P. R. China

N. L.
School of Electronics Information and Electrical Engineering, Shanghai Jiao Tong University, Shanghai, 200240, P. R. China

†These authors contributed equally to this work.

This part included:

1. Materials and Methods
2. Figure S1-S12
3. Table S1-S11

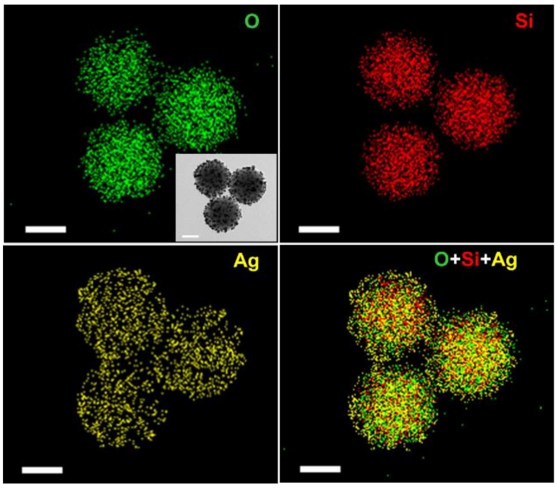

Figure S1. Elemental mapping of SiO_2@Ag nanoshells shown with oxygen (O) element in green, silicon (Si) element in red and silver (Ag) in yellow. Scale bar: 100 nm.

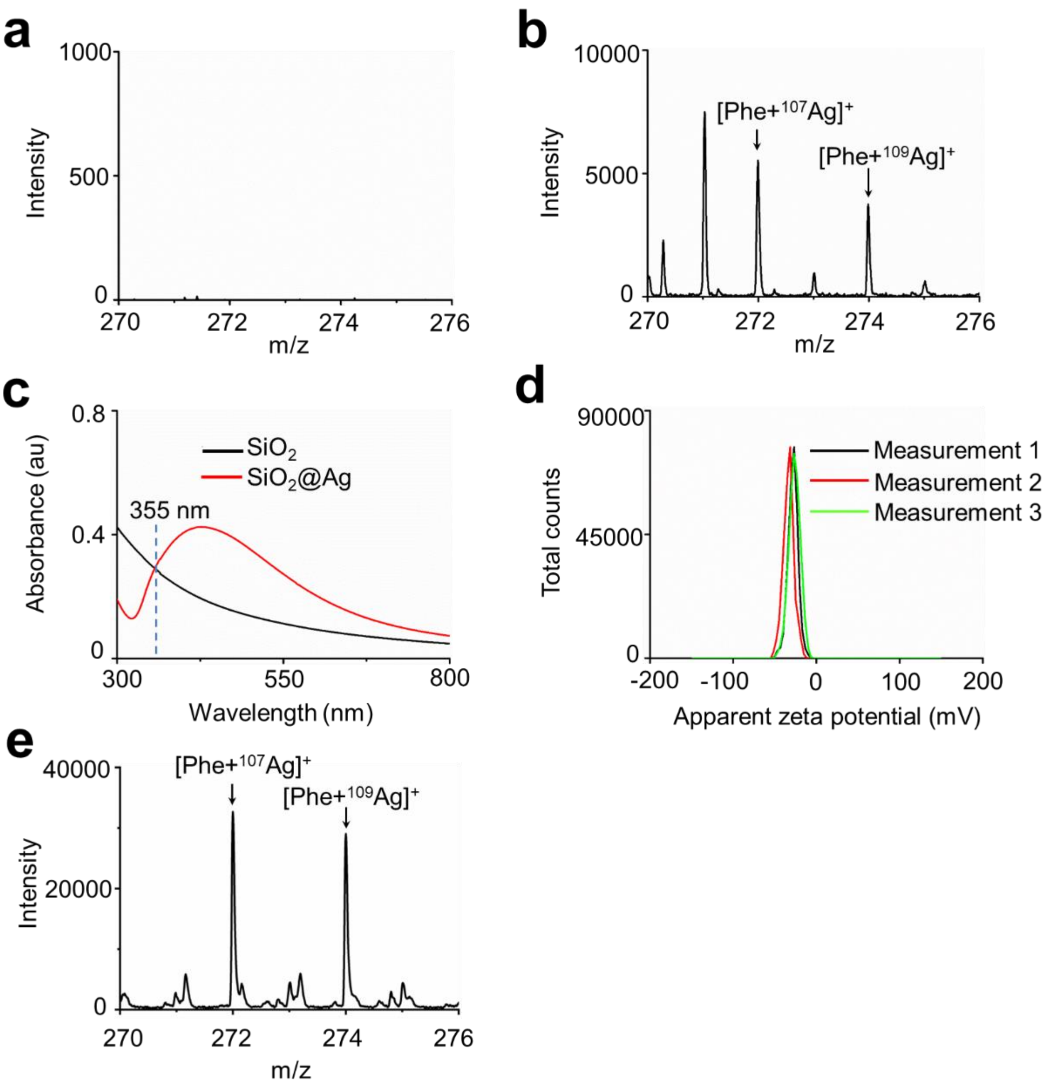

Figure S2. The mass spectra of phenylalanine (Phe) (1ng/μL) directly dropped on the polish plate without (a) and with (b) SiO_2@Ag nanoshells as matrix. The ultraviolet-visible (UV-Vis) spectroscopy (c) and apparent zeta potential (d) of SiO_2@Ag nanoshells. (e) Mass spectra of Phe in a DBS sample of PKU patient. The dashed line (in Figure S2c) indicated a wavelength of 355 nm (the wavelength of Nd:YAG laser equipped in LDI MS system). Zeta potential was recorded by three independent measurements.

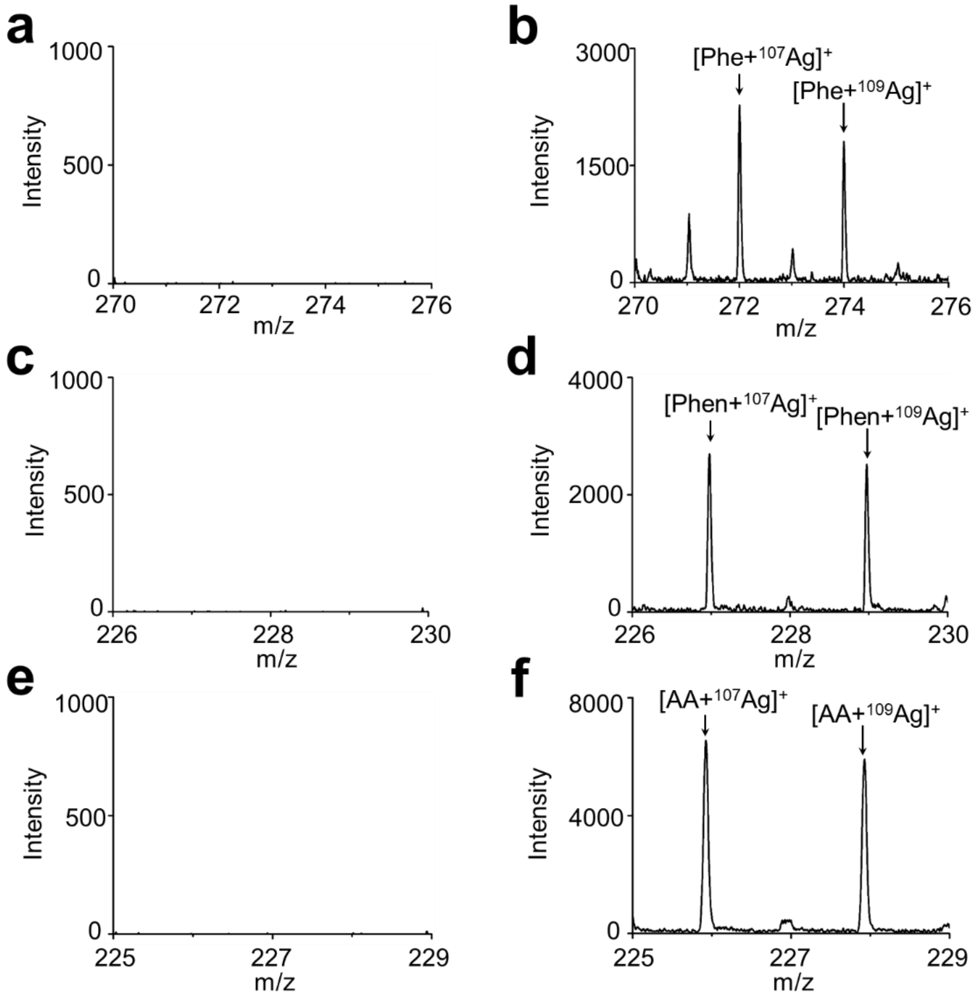

Figure S3. Mass spectra of (a, b) 1 ng μL^{-1} Phe, (c, d) 1 ng μL^{-1} phenylacetaldehyde (Phen) and (e, f) 10 ng μL^{-1} aminomalonic acid (AA) directly dropped on the polish plate without matrix (a, c, e) and using SiO_2@Ag as matrix (b, d, f).

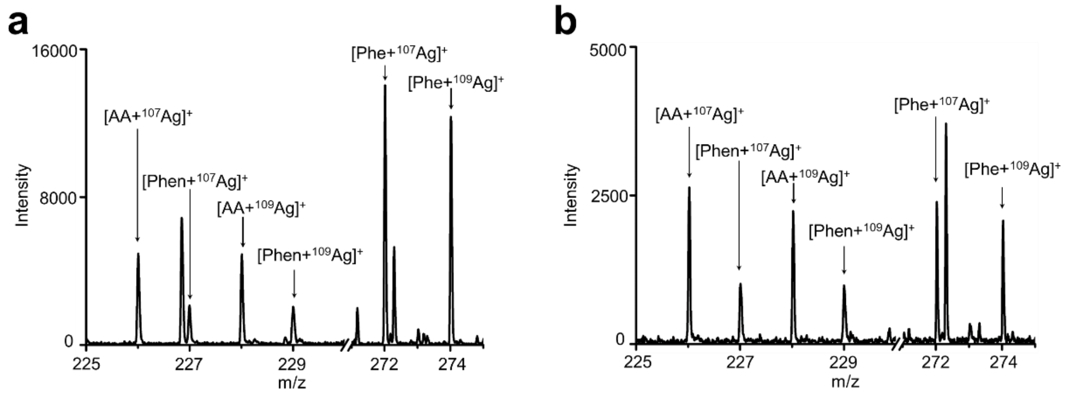

Figure S4. Mass spectra of 10 ng μL^{-1} Phe, Phen and AA in (a) 0.5 M NaCl and (b) 5 mg mL^{-1} BSA solutions using SiO_2@Ag as the matrix.

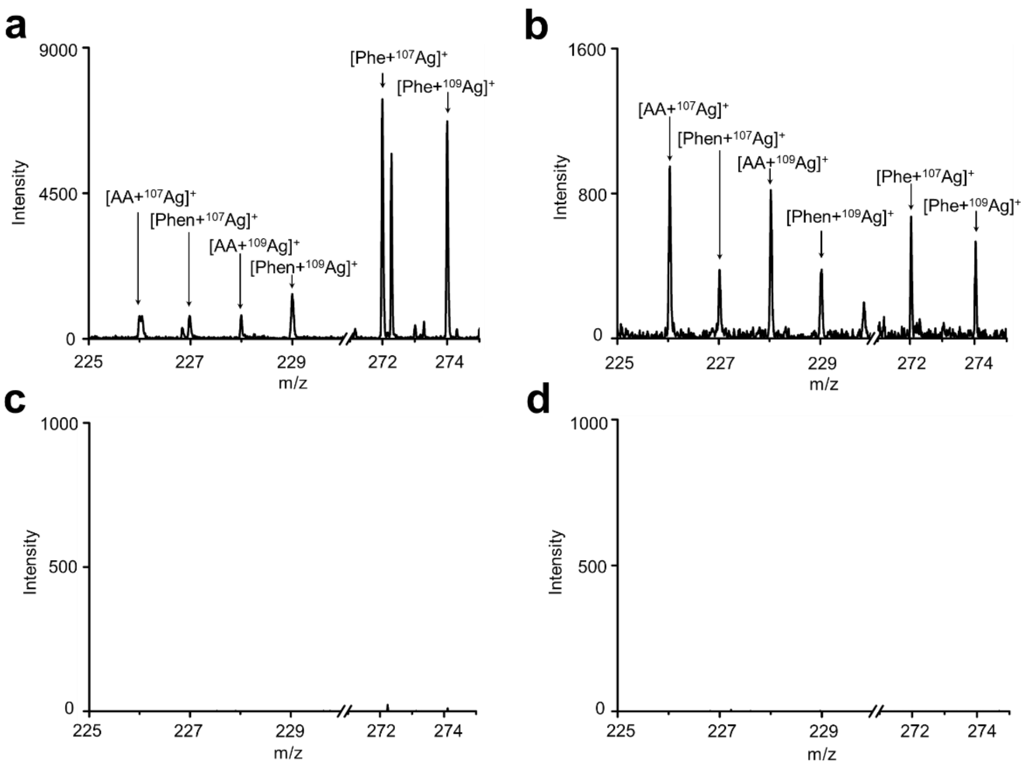

Figure S5. Mass spectra of 10 ng μL^{-1} Phe, Phen and AA in (a, c) 0.5 M NaCl and (b, d) 5 mg mL^{-1} BSA solutions using Ag nanoparticles (a, b) and SiO_2 nanoparticles (c, d) as the matrix, respectively.

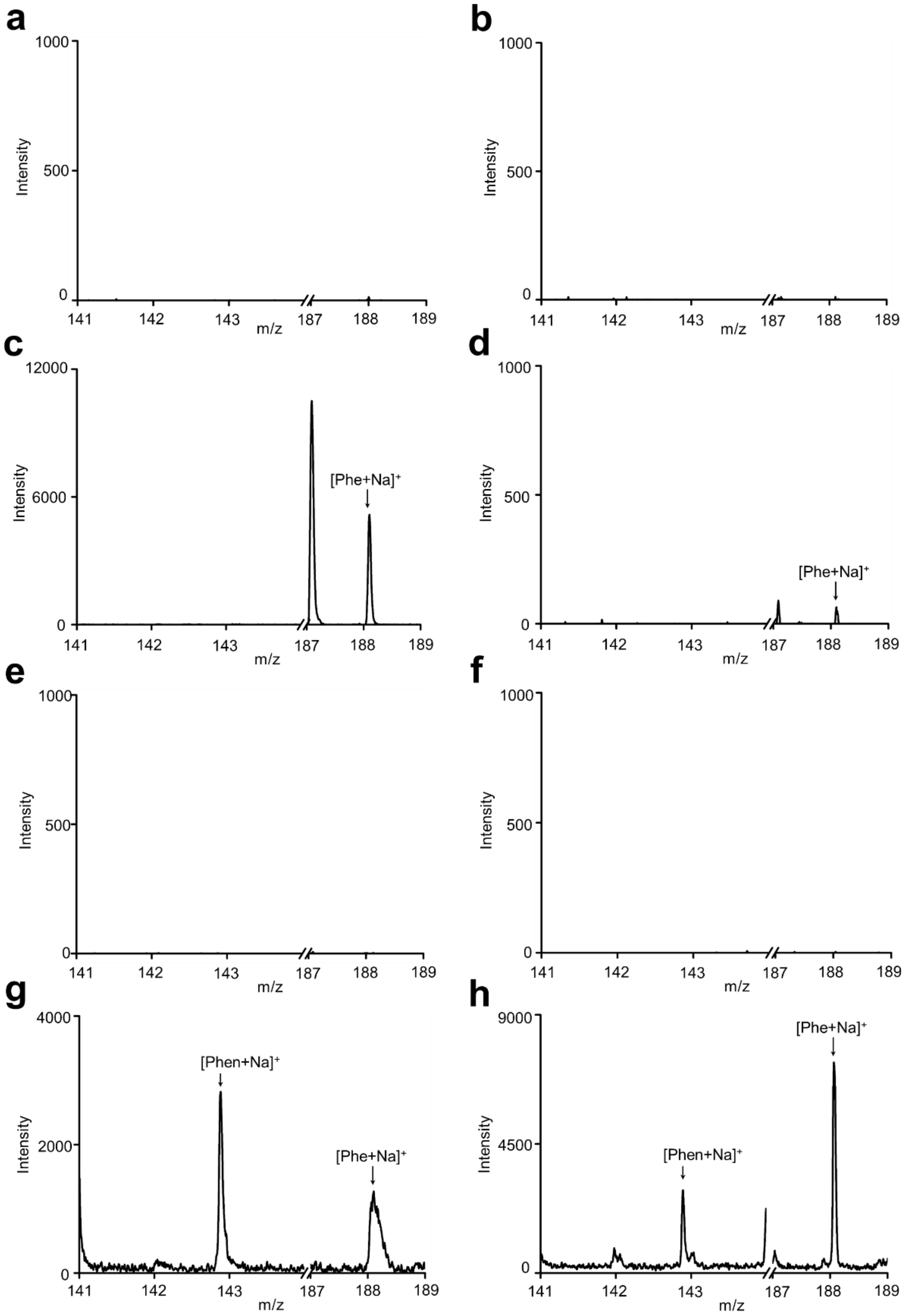

Figure S6. Mass spectra of 10 ng μL^{-1} Phe, Phen and AA in (a, c, e, g) 0.5 M NaCl and (b, d, f, h) 5 mg mL^{-1} BSA solutions using DHB (a, b), CHCA (c, d), Au nanoparticles (e, f) and magnetic nanoparticles (g, h) as the matrix, respectively.

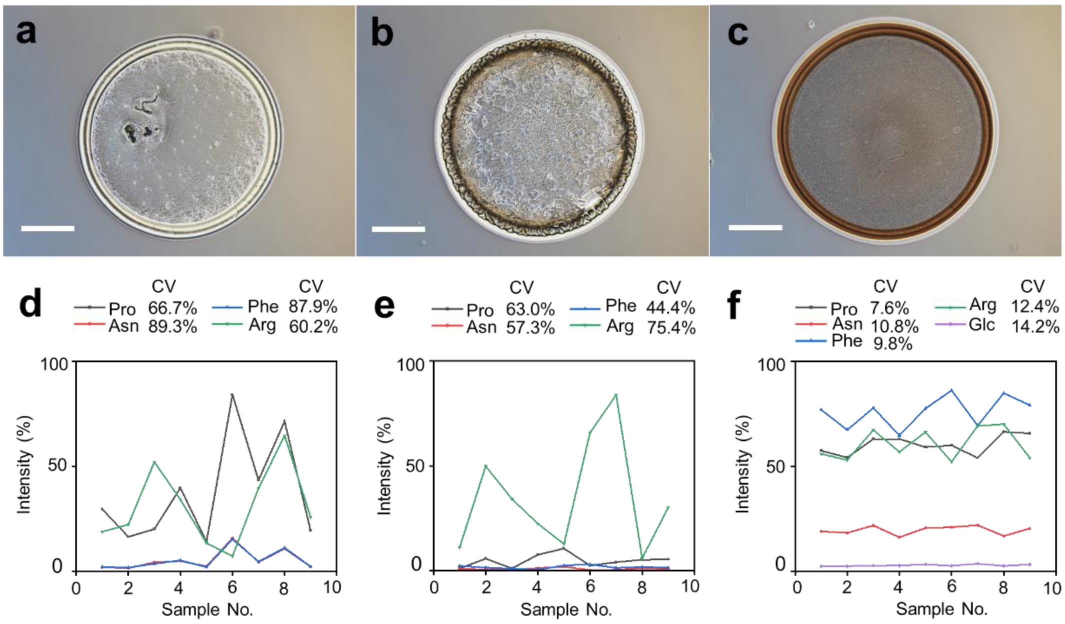

Figure S7. Optical microscope images of matrix-analyte crystallization using (a) CHCA, (b) DHB and (c) SiO_2@Ag nanoparticles as matrices. The scale bars were 500 μm. Coefficient of variances (CVs) distribution of different metabolites (proline (Pro), asparagine (Asn), phenylalanine (Phe), arginine (Arg), and glucose (Glc)) detected by (d) CHCA, (e) DHB and (f) SiO_2@Ag nanoparticles assisted LDI MS. We can't observe the Glc adducted peaks when CHCA and DHB as matrix. The results come from 9 independent experiments.

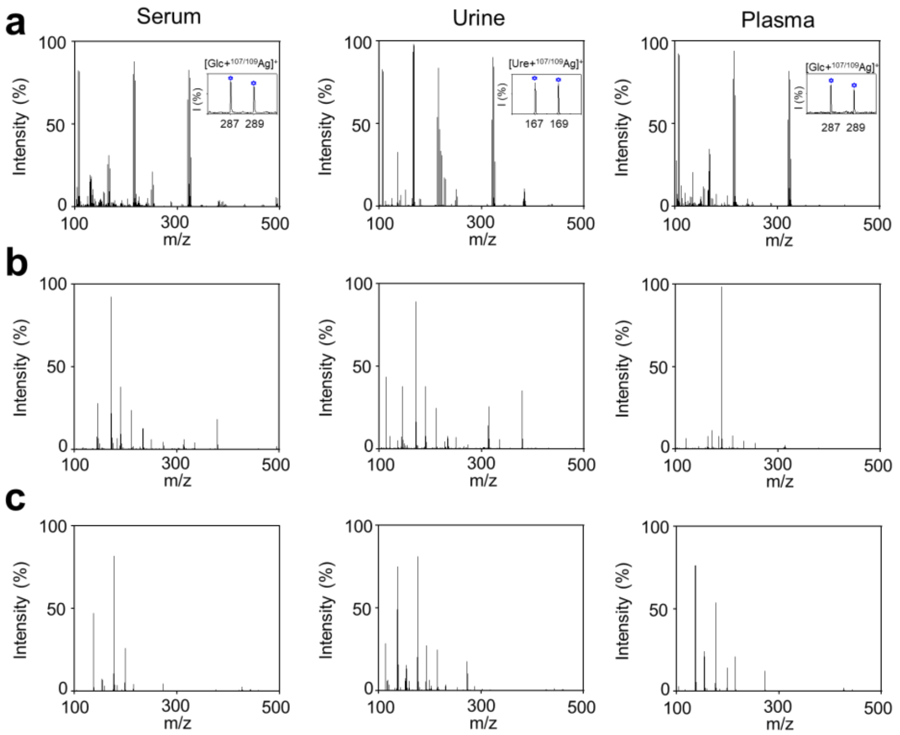

Figure S8. Mass spectra of metabolites in serum, urine and plasma detected by (a) SiO_2@Ag, (b) CHCA, and (c) DHB assisted LDI MS. The inset figures in Figure S8a show the Ag-adducted peaks of typical urea (Ure) metabolite in urine and typical glucose (Glc) metabolite in serum and plasma.

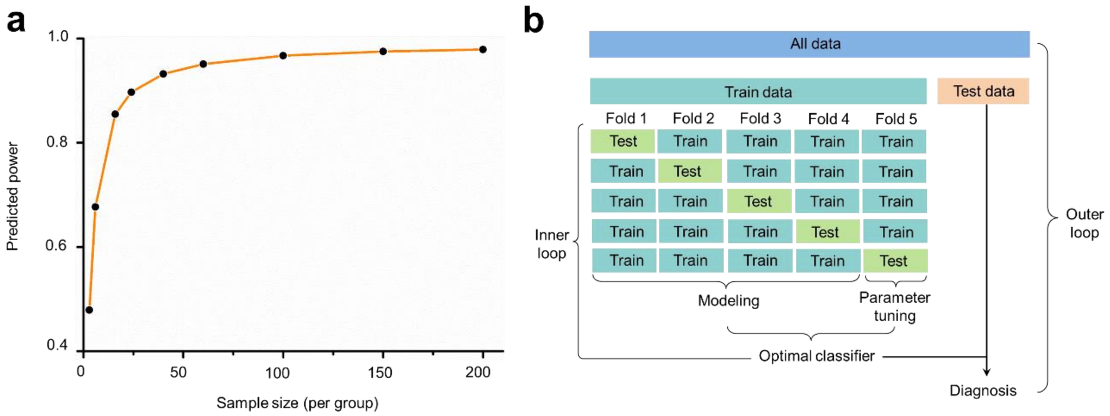

Figure S9. (a) Power analysis based on DBS metabolic profiles, including 10 samples (5/5, healthy control/PKU) for calculating the required sample number for machine learning. A power of 0.90 could be obtained with the sample number of 48 (24/24, healthy control/PKU) at a false discovery rate of 0.10. (b) Schematic workflow for the construction of classification models, including an inner loop to tune the parameters for the optimal classifier and outer loop to evaluate the diagnostic performance.

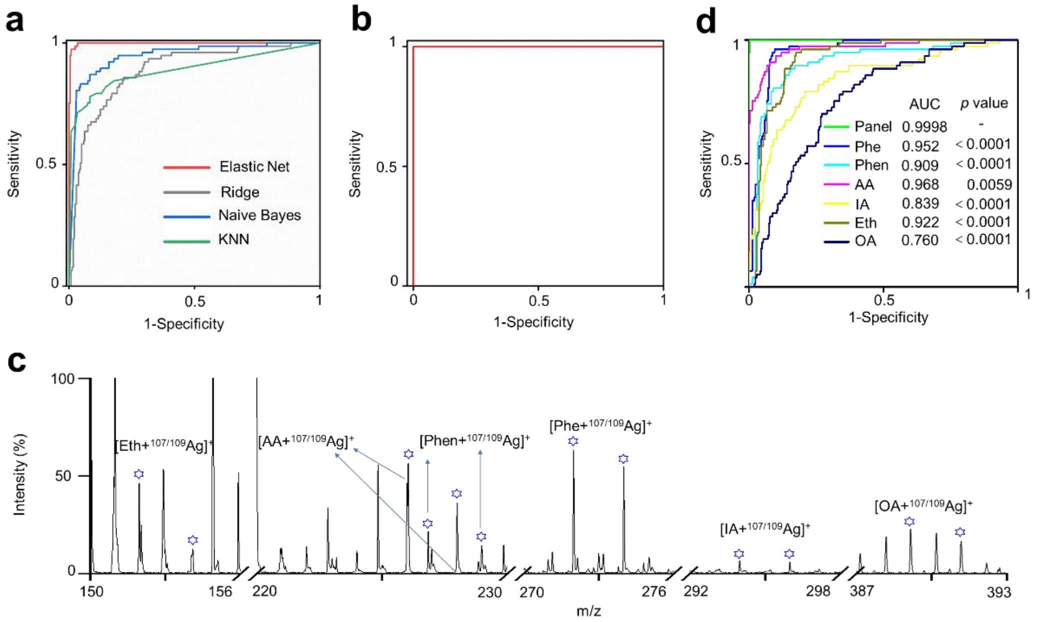

Figure S10. (a) Receiver operating characteristic (ROC) curves using Elastic Net, Ridge, Naive Bayes and KNN to distinguish PKU patients from healthy controls in the discovery cohort for PKU screening. (b) ROC curve of Elastic Net as classifier to discriminate subtype PAH from BH4 in the validation cohort including 9 samples (PAH/BH4, 5/4). (c) The Ag-adducted peaks of typical features including phenylalanine (phe), phenylacetaldehyde (Phen), aminomalonic acid (AA), indoleacrylic acid (IA), ethylamine (Eth) and oleic acid (OA) in dried blood spot. (d) Receiver operating characteristic (ROC) curves using single biomarker and 6-biomarker panel to distinguish PKU patients from healthy controls in the discovery cohort for PKU diagnosis.

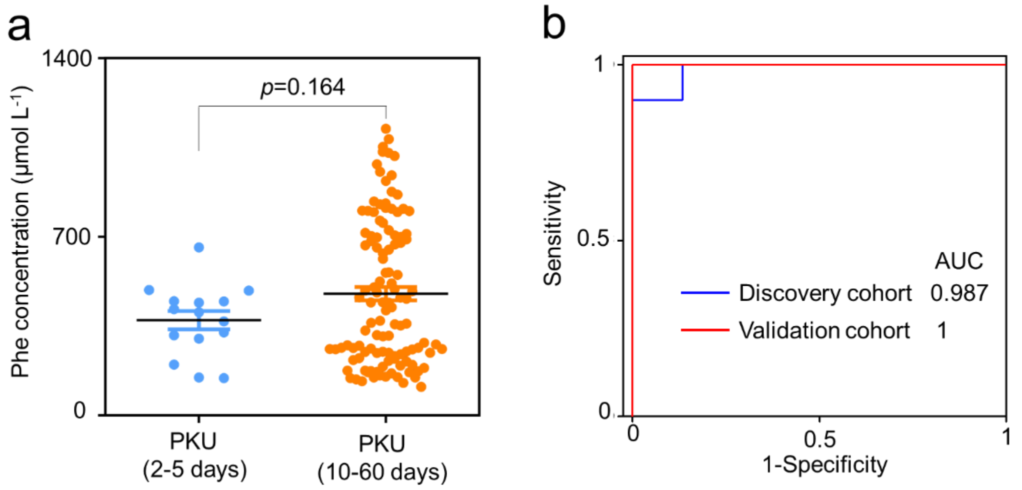

Figure S11. (a) The difference of Phe concentration of 2-5 day-old PKU (15 patients) and 10-60 day-old PKU patients (115 patients). (b) ROC curve produced by sparse learning for distinguishing 2-5 day-old PKU patients from healthy controls in the discovery cohort and validation cohort.

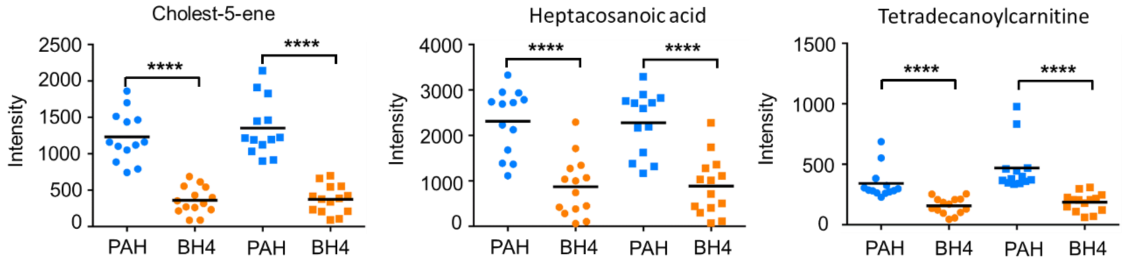

Figure S12. Scatter diagram of 3 key differential metabolites with $[M+^{107}Ag]^+$ and $[M+^{109}Ag]^+$ for PKU subtype identification (PAH/BH4, 13/14), including cholest-5-ene, tetradecanoylcarnitine and heptacosanoic acid. **** represented $p < 0.0001$.

Table S1. Clinical characteristics of healthy controls and PKU patients for discovery and validation cohorts

Characteristics	Discovery cohort			Validation cohort		
	Healthy control n=240	PKU n=77	*p* value	Healthy control n=730	PKU n=24	*p* value
Sex			0.66[a]			0.926[b]
Male	124	42	-	372	12	-
Female	116	35	-	358	12	-
Age	All subjects were newborns between 2 and 60 days old					

[a, b] *p* value was calculated by $\chi 2$ test.

Table S2. The classification performance of different algorithms on discovery cohort for PKU screening

Model	Discovery cohort			
	AUC (95% CI)	*p* value[a]	Sensitivity	Specificity
Ridge	0.887 (0.847-0.920)	< 0.0001	0.857	0.783
Naive Bayes	0.947 (0.916-0.969)	0.0003	0.870	0.917
KNN	0.890 (0.850-0.922)	< 0.0001	0.779	0.917
Elastic Net	0.998 (0.985-1)	-	0.985	0.995

[a]DeLong test results showed the significant level of ROC curves of Ridge, Naive Bayes and KNN compared with Elastic Net.

Table S3. The sensitivity and specificity optimized by maximizing Youden index, closest to (0, 1) corner in ROC curve, equal sensitivity and specificity in different classification models

Model	Discovery cohort					
	Youden index		(0, 1) corner		Equal Sen and Spe	
	Sen[a)]	Spe[b)]	Sen	Spe	Sen	Spe
Ridge	0.857	0.783	0.857	0.783	0.818	0.808
KNN	0.779	0.917	0.818	0.854	0.844	0.821
Naive Bayes	0.870	0.917	0.870	0.917	0.883	0.875
Elastic Net	1	0.967	0.985	0.995	0.974	0.975

a) Sen is short for sensitivity.
b) Spe is short for specificity.

Table S4. Sample information of 15 PKU patients with DBS samples collected in 2-5 days after birth

Samples	Sex	Age (day)	Concentration of Phe (μmol L^{-1})
1	Female	3	489.42
2	Male	3	325.74
3	Male	3	416.79
4	Male	3	447.31
5	Female	3	447.00
6	Male	4	491.73
7	Male	3	404.19
8	Male	2	368.67
9	Female	3	300.71
10	Female	3	442.22
11	Male	3	199.00
12	Female	5	658.85
13	Male	3	146.46
14	Male	3	313.83
15	Female	3	148.50

Table S5. Comparison of the concentration of Phe in 10-60 day-old PKU and 2-5 day-old PKU patients

Samples	Number	Age	Mean of Phe concentration (μmol L^{-1})	*p* value[a)]
PKU 1	115	10-60 days	476.8	0.164
PKU 2	15	2-5 days	373.4	

[a)] *p* value was calculated by t-test.

Table S6. Clinical characteristics of healthy controls and PKU patients with age of 2-5 days

Characteristics	Discovery cohort			Validation cohort		
	Control n=15	PKU n=10	*p* value	Control n=10	PKU n=5	*p* value
Sex			0.87[a)]			0.14[b)]
Male	8	5	-	4	4	-
Female	7	5	-	6	1	-

[a, b)] *p* value was calculated by $\chi 2$ test.

Table S7. Sample information of 27 PKU patients for subtype identification (including 13 PAH and 14 BH4 patients)

Sample	Metabolites in DBS		Metabolites in urine		
	Phe (μmol/L)	Phe/Tyr	N (mmol/molCr)	B (mmol/molCr)	B%
(PAH)1	683.404	14.61	0.87	0.35	28.57
(PAH)2	442.68	4.847	0.91	0.59	39.45
(PAH)3	164.732	2.687	0.45	0.87	65.72
(PAH)4	309.862	5.435	0.47	0.21	30.41
(PAH)5	666.939	11.034	1.30	0.36	21.77
(PAH)6	258.392	2.726	10.87	0.28	2.55
(PAH)7	613.528	8.639	2.46	0.68	21.61
(PAH)8	828.082	9.664	0.57	0.30	34.58
(PAH)9	216.638	4.03	0.52	1.64	75.85
(PAH)10	151.906	2.566	1.99	0.35	14.84
(PAH)11	261.808	2.516	0.51	0.14	21.19
(PAH)12	250.35	1.974	0.64	0.16	20.00
(PAH)13	146.38	2.292	0.98	0.71	42.08
(BH4)1	312.458	2.609	3.97	0.15	3.53
(BH4)2	703.503	10.646	6.20	0.09	1.40
(BH4)3	1052.288	23.696	4.39	0.08	1.79
(BH4)4	195.14	3.559	5.95	0.27	4.33
(BH4)5	284.372	2.387	3.00	0.12	3.86
(BH4)6	127.273	2.46	2.36	0.10	4.14
(BH4)7	877.04	13.343	4.70	0.12	2.56
(BH4)8	370.91	5.2	5.08	0.11	2.03
(BH4)9	170.853	1.721	1.69	0.09	5.10
(BH4)10	697.212	7.842	5.69	0.20	3.37
(BH4)11	634.446	6.315	2.73	0.09	3.14
(BH4)12	488.95	7.033	5.60	0.27	4.60
(BH4)13	517.507	6.336	2.87	0.08	2.63
(BH4)14	670.014	12.02	7.15	0.14	1.96

Table S8. Sample information of 20 healthy controls for subtype identification

Sample	Metabolites in DBS	
	Phe (μmol/L)	Phe/Tyr
(Healthy)1	64.952	0.899
(Healthy)2	55.842	1.132
(Healthy)3	63.64	0.838
(Healthy)4	61.985	0.883
(Healthy)5	49.941	0.588
(Healthy)6	44.296	0.751
(Healthy)7	61.136	0.69
(Healthy)8	64.872	1.376
(Healthy)9	61.159	1.358
(Healthy)10	58.941	0.812
(Healthy)11	58.216	0.915
(Healthy)12	54.393	0.45
(Healthy)13	56.621	0.624
(Healthy)14	50.16	0.826
(Healthy)15	75.616	0.684
(Healthy)16	51.663	0.883
(Healthy)17	64.564	0.913
(Healthy)18	54.916	0.968
(Healthy)19	49.873	0.88
(Healthy)20	67.616	1.424

Table S9. Metabolites selected for differentiating PKU from healthy controls

Metabolite	Molecular weight (Da)	Adduction signals		Frequency[a]	Coefficient[b]	*p* value[c]	AUC[d]
		Adduction	m/z (Da)				
Phe	165.08	$[M+^{107}Ag]^+$	271.98	94	0.0107	4.31E-186	0.937
		$[M+^{109}Ag]^+$	273.98	100	0.1988	6.16E-244	0.967
Phen	120.06	$[M+^{107}Ag]^+$	226.96	100	0.1290	3.43E-209	0.901
		$[M+^{109}Ag]^+$	228.96	100	0.2042	1.04E-191	0.903
AA	119.02	$[M+^{107}Ag]^+$	225.93	100	0.4037	8.35E-236	0.949
		$[M+^{109}Ag]^+$	227.93	100	0.1531	8.45E-235	0.930
IA	187.06	$[M+^{107}Ag]^+$	293.96	95	0.0449	3.19E-184	0.902
		$[M+^{109}Ag]^+$	294.97	84	0.0090	7.55E-179	0.908
Eth	45.06	$[M+^{107}Ag]^+$	151.96	100	-0.4920	3.63E-117	0.895
		$[M+^{109}Ag]^+$	153.96	100	-0.0733	8.69E-26	0.685
OA	282.26	$[M+^{107}Ag]^+$	389.16	100	-0.1984	3.29E-20	0.643
		$[M+^{109}Ag]^+$	391.16	93	-0.0530	5.25E-15	0.619

[a]Frequency referred to selection probability by optimized classifier for PKU diagnosis in 100 models.
[b]Coefficient referred to the statistical weight calculated as the sparsity constraints of the diagnostic classifier.
[c]*p* value was acquired for healthy controls and PKU through two-sided Student's t-test. [d]AUC was acquired by ROC curve analysis using the individual biomarker.

Table S10. Tandem MS summary of $^{107}Ag^{+}$- and $^{109}Ag^{+}$-adducted biomarkers

Metabolite adduction[a]	Fragments	Mass to charge ratio (m/z, Da)	Standard solution[b]	Clinical sample[c]
$[Phe+^{107}Ag]^+$	$[C_6H_2NO_2+^{107}Ag]^+$	226.91	√	√
	$[C_9H_{11}NO_2+^{107}Ag]^+$	271.98	√	√
$[Phe+^{109}Ag]^+$	$[C_9H_{11}NO_2+H]^+$	166.09	√	√
	$[C_6H_2NO_2+^{109}Ag]^+$	228.91	√	√
	$[C_9H_9O_2+^{109}Ag]^+$	257.96	√	√
	$[C_9H_{11}NO_2+^{109}Ag]^+$	273.98	√	√
$[Phen+^{107}Ag]^+$	$[C_6H_3+^{107}Ag]^+$	181.93	√	√
	$[C_6H_5+^{107}Ag]^+$	183.94	√	√
	$[C_6H_3O+^{107}Ag]^+$	197.92	√	√
	$[C_8H_8O+^{107}Ag]^+$	226.96	√	√
$[Phen+^{109}Ag]^+$	$[C_4H_7O+^{109}Ag]^+$	179.95	√	√
	$[C_6H_3+^{109}Ag]^+$	183.93	√	√
	$[C_6H_5+^{109}Ag]^+$	185.94	√	√
	$[C_6H_3O+^{109}Ag]^+$	199.92	√	√
	$[C_8H_8O+^{109}Ag]^+$	228.96	√	√
$[AA+^{107}Ag]^+$	$[CH_6N+^{107}Ag]^+$	138.95	√	√
	$[CHO_2+^{107}Ag]^+$	151.90	√	√
	$[CH3O_2+^{107}Ag]^+$	153.92	√	√
	$[C_2H_4NO_2+^{107}Ag]^+$	180.93	√	√
	$[C_2H_6NO_2+^{107}Ag]^+$	182.94	√	√
	$[C_3H_5NO_4+^{107}Ag]^+$	225.93	√	√
$[AA+^{109}Ag]^+$	$[CH_4N+^{109}Ag]^+$	138.94	√	√
	$[CH_6N+^{109}Ag]^+$	140.95	√	√
	$[C_2H_4NO_2+^{109}Ag]^+$	182.93	√	√
	$[C_2H_6NO_2+^{109}Ag]^+$	184.94	√	√
	$[C_3H_5NO_4+^{109}Ag]^+$	227.93	√	√
$[IA+^{107}Ag]^+$	$[C_6H_5+^{107}Ag]^+$	183.94	√	√
	$[C_6H_7+^{107}Ag]^+$	185.96	√	√
	$[C_{10}H_8N+^{107}Ag]^+$	248.97	√	√
	$[C_{10}H_9N+^{107}Ag]^+$	249.98	√	√

	$[C_{11}H_9NO_2+^{107}Ag]^+$	293.97	√	√
$[IA+^{109}Ag]^+$	$[C_7H_3N+^{109}Ag]^+$	209.93	√	√
	$[C_{10}H_9N+^{109}Ag]^+$	251.98	√	√
	$[C_{11}H_8NO+^{109}Ag]^+$	278.96	√	√
	$[C_{11}H_9NO_2+^{109}Ag]^+$	295.97	√	√
$[Eth+^{107}Ag]^+$	$[C_2H_5+^{107}Ag]^+$	135.94	√	√
	$[C_2H_7N+^{107}Ag]^+$	151.96	√	√
$[Eth+^{109}Ag]^+$	$[C_2H_5+^{109}Ag]^+$	137.94	/	/
	$[C_2H_7N+^{109}Ag]^+$	153.96	√	√
$[OA+^{107}Ag]^+$	$[C_7H_{11}O_2+^{107}Ag]^+$	233.98	√	√
	$[C_{11}H_{19}O_2+^{107}Ag]^+$	290.04	√	√
	$[C_{12}H_{21}O_2+^{107}Ag]^+$	304.06	√	√
	$[C_{17}H_{32}+^{107}Ag]^+$	343.15	√	√
	$[C_{15}H_{27}O_2+^{107}Ag]^+$	346.11	√	√
	$[C_{18}H_{34}O_2+^{107}Ag]^+$	389.16	√	√
$[OA+^{109}Ag]^+$	$[C_{11}H_{19}O_2+_{109}Ag]_+$	292.04	√	√
	$[C_{12}H_{21}O_2+^{109}Ag]^+$	306.06	√	√
	$[C_{17}H_{32}+^{109}Ag]^+$	345.15	√	√
	$[C_{15}H_{27}O_2+^{109}Ag]^+$	348.11	√	√
	$[C_{18}H_{34}O_2+^{109}Ag]^+$	391.16	√	√

[a]Metabolite adduction referred to the adduction peaks of $[M+^{107}Ag]^+$ and $[M+^{109}Ag]^+$ for 6 biomarkers including Phe, Phen, AA, IA, Eth, and OA.

[b]Standard solution was prepared by dispersing each biomarker molecule in water with concentration of 1mg/mL. √ referred to that the fragment was detectable. / referred to that the fragment was undetectable.

[c]PKU samples were used for validating Phe, Phen, AA, IA. Healthy control samples were used for validating Eth, and OA. √ referred to that the fragment was detectable. / referred to that the fragment was undetectable.

Table S11. Metabolites selected for differentiating PAH from BH4

Metabolite	Molecular weight (Da)	Adduction signals		Frequency^{a}	Coefficient^{b}	*p* value^{c}	AUC^{d}
		Adduction	m/z (Da)				
Cholest-5-ene	370.36	$[M+^{107}Ag]^+$	477.26	100	-0.4405	6.07E-31	0.979
		$[M+^{109}Ag]^+$	479.26	100	-0.4303	8.45E-32	0.982
Heptacosanoic acid	410.41	$[M+^{107}Ag]^+$	517.32	100	-0.1952	3.30E-19	0.897
		$[M+^{109}Ag]^+$	519.32	100	-0.1858	6.11E-19	0.895
Tetradecanoylcarnitine	372.31	$[M+^{107}Ag]^+$	478.21	100	-0.1692	4.27E-18	0.945
		$[M+^{109}Ag]^+$	480.21	100	-0.2649	1.17E-19	0.983

^{a}Frequency referred to selection probability by optimized classifier for subtype identification in 100 models.
^{b}Coefficient referred to the statistical weight calculated as the sparsity constraints of the diagnostic classifier.
^{c}*p* value was acquired for identification of PAH and BH4 through two-sided Student's t-test.
^{d}AUC was acquired by ROC curve analysis using the individual biomarker.
